# Characterization of intrinsically disordered regions in proteins informed by human genetic diversity

**DOI:** 10.1371/journal.pcbi.1009911

**Published:** 2022-03-11

**Authors:** Shehab S. Ahmed, Zaara T. Rifat, Ruchi Lohia, Arthur J. Campbell, A. Keith Dunker, M. Sohel Rahman, Sumaiya Iqbal

**Affiliations:** 1 Department of Computer Science and Engineering, Bangladesh University of Engineering and Technology, ECE Building, West Palashi, Dhaka-1205, Bangladesh; 2 Cold Spring Harbor Laboratory, Cold Spring Harbor, New York, United States of America; 3 Center for the Development of Therapeutics, Broad Institute of MIT and Harvard, Cambridge, Massachusetts, United States of America; 4 Stanley Center for Psychiatric Research, Broad Institute of MIT and Harvard, Cambridge, Massachusetts, United States of America; 5 Center for Computational Biology and Bioinformatics, Department of Biochemistry and Molecular Biology, Indiana University School of Medicine, Indianapolis, Indiana, United States of America; 6 Program in Medical and Population Genetics, Broad Institute of MIT and Harvard, Cambridge, Massachusetts, United States of America; 7 Analytic and Translational Genetics Unit, Massachusetts General Hospital, Boston, Massachusetts, United States of America; Fox Chase Cancer Center, UNITED STATES

## Abstract

All proteomes contain both proteins and polypeptide segments that don’t form a defined three-dimensional structure yet are biologically active—called intrinsically disordered proteins and regions (IDPs and IDRs). Most of these IDPs/IDRs lack useful functional annotation limiting our understanding of their importance for organism fitness. Here we characterized IDRs using protein sequence annotations of functional sites and regions available in the UniProt knowledgebase (“UniProt features”: active site, ligand-binding pocket, regions mediating protein-protein interactions, etc.). By measuring the statistical enrichment of twenty-five UniProt features in 981 IDRs of 561 human proteins, we identified eight features that are commonly located in IDRs. We then collected the genetic variant data from the general population and patient-based databases and evaluated the prevalence of population and pathogenic variations in IDPs/IDRs. We observed that some IDRs tolerate 2 to 12-times more single amino acid-substituting missense mutations than synonymous changes in the general population. However, we also found that 37% of all germline pathogenic mutations are located in disordered regions of 96 proteins. Based on the observed-to-expected frequency of mutations, we categorized 34 IDRs in 20 proteins (DDX3X, KIT, RB1, etc.) as intolerant to mutation. Finally, using statistical analysis and a machine learning approach, we demonstrate that mutation-intolerant IDRs carry a distinct signature of functional features. Our study presents a novel approach to assign functional importance to IDRs by leveraging the wealth of available genetic data, which will aid in a deeper understating of the role of IDRs in biological processes and disease mechanisms.

## Author summary

Intrinsically disordered regions (IDRs) in proteins are typically not considered to be functionally as important as the structured parts. However, it is becoming evident that both structured and disordered regions are essential for the repertoire of protein functions. Nevertheless, most of these largely flexible and functionally dynamic protein regions remain uncharacterized. Here, informed by human genetic diversity (i.e., genetic variations from the general population and patients), we identified the IDRs that are more frequently mutated in patients than in relatively healthy individuals, and further show that they carry a set of characteristic functional features. This approach provides a different and effective means to identify unannotated disordered protein segments that are biologically important and lead to pathogenesis upon mutation.

## Introduction

In contrast to the standard protein structure-function paradigm, it is now recognized that many proteins, in their entirety or partly in regions, lack a defined three-dimensional (3D) structure under physiological conditions, but still carry out a wide range of cellular functions [1,2]. These biologically active, dynamic proteins and regions in proteins are known as intrinsically disordered proteins (IDPs) or regions (IDRs) [3]. Several sequence-based (i.e., physicochemical) and structural properties of IDPs and IDRs are now well-established, such as high net-charge, low hydrophobicity, high propensity to form pliable coils, depletion of aromatic residues, low sequence complexity [4–6]. While different combinations of these properties can hint to disordered regions’ functional flavors and preferential conformations [7,8], biophysical and biochemical experiments are essential for a reliable characterization of their functions. However, experimental methods are mostly low-throughput and impose many technical challenges due to these proteins’ disordered nature and tendency to be involved in promiscuous and transient interactions [9,10]. Bioinformatics and computational biology methods are well suited to gain information about IDPs [11–15]. In light of the growing success of predictive methods in determining the commonness of IDRs and in detecting IDRs and their functions, a biennial experiment inspired by the critical assessment of protein structure prediction (CASP) for the benchmarking of intrinsic disorder (CAID) has been established [16].

A rich collection of studies is available documenting the varied functional features of IDPs/IDRs that complement the functional repertoire of structured proteins [17,18]. It has been shown that disordered regions in proteins predominantly contain molecular-recognition features (MoRFs) [19], post-translational modification (PTMs) sites [17], short linear peptide motifs [13], protein- and DNA-binding regions [20–22], and flexible linkers or spacers [23,24]. Experimental annotation of the function of IDRs being not scalable, the use of machine learning algorithms played a complementary role in the prediction of their function [25–27]. Another way of characterizing the function of protein regions is to utilize the annotations of “sequence features” available in the UniProt knowledgebase repository [28] in terms of sites of biological interest in proteins, e.g., active sites, metal-binding sites, domains, residues involved in molecular processing. Studies have demonstrated that proteins from different functional classes show variable enrichment and depletion of these features in proteins’ 3D structures [29]. Notably, no study has been published to date that systematically determines the association between these UniProt sequence features and disordered regions of proteins, which could identify the functional elements that are ubiquitously present in IDRs.

Concomitantly with the investigation of the functional role of IDPs, their abundance and evolutionary characteristics have also been extensively studied, mostly through cross-species sequence alignments and structure comparisons [30,31]. Out of disordered regions of different lengths, long IDRs reportedly display a high evolutionary rate [32,33], yet preserve their function. At the same time, It has been demonstrated that genetically and environmentally altered IDPs lead to many pathological conditions through various mechanisms: perturbation of protein-protein interactions, change of the sequence’s chemico-physical character and disorder propensity, leading to aggregation and distortion of PTM sites, and thereby causing missignaling, misregulations and susceptibility to pathogens, etc. [9,18]. Recent high-throughput genome and exome sequencing projects have enabled the detection of human genetic variants at an unprecedented scale [34]. Subsequently, much attention has been put on the characterization of the structural regions of proteins that are intolerant to genetic variations [29,35]; however, much less is known about the disordered regions. The available genetic variation data, along with the IDR annotation in multiple databases such as DisProt [36], MobiDB [37], IDEAL [38], and functional site annotations of protein sequences in the UniProt database [28], now calls for a data-driven approach to annotate and characterize IDRs that are intolerant to genetic variations (or mutations). Progress towards identification of IDRs that are intolerant to mutations and these regions’ features (i.e., functional sites of interest) will advance our understanding of the disease-vulnerable properties of IDRs, their role in disease etiology, and will aid in designing drugs against IDPs.

With this study, we first sought to characterize the experimentally verified disordered regions of intrinsically disordered proteins in human (collected from DisProt database) using the residue position-specific annotations of sites available in the UniProt (referred to as "UniProt features"). The rationale behind this approach is that the disorder propensity of protein regions is encoded in their residue composition, which is noticeably different from that of the structured domain [4,39]. We therefore hypothesize that disordered regions are likely to carry a unique set of UniProt features compared to the rest of the protein. Then by comparing the frequency of “population” and “pathogenic” genetic variations in IDRs, we identified disordered regions that are relatively more or less intolerant to mutations. Further characterization of mutation-intolerant IDRs using the same set of UniProt features showed that mutation-intolerant IDRs carry a distinct set of properties compared to those, that are relatively tolerant. To the best of our knowledge, this work is the first of its kind for IDPs especially in terms of the data analyzed: Variants from gnomAD [40] and ClinVar [41] databases and feature annotations from UniProt database [28].

## Results

This study has been performed on human intrinsically disordered proteins (IDPs) that were annotated with disorder information, i.e., whether a residue/region is disordered and its category in the DisProt database (release 2020_06) [36], and residue position-specific “UniProt feature” information, indicating sites of biological interest in proteins, in the UniProt database (release 2020_02) [28]. We investigated 561 out of 567 human IDPs that were annotated in both DisProt and UniProt (*Materials and Methods*). These 561 proteins contain 981 disordered regions (IDRs) comprising 58,993 disordered residues or DRs (**S1 Table**), while the rest are referred to as non-annotated residues, or NRs.

### UniProt features associated with disordered protein residues

To systematically identify the “UniProt features” associated with disordered residues, we computed the fraction of disordered residues as well as their association with each feature compared to non-annotated residues for 561 IDPs. We investigated a set of twenty-five features corresponding to different sites of interest in proteins according to the UniProt database. Statistical associations between residue-wise features and residue types (disordered vs. non-annotated) were quantified using the two-tailed Fisher’s Exact test. An odds ratio (OR) > 1.0 and corrected p-value, *q* < 0.05 indicates features enriched in IDRs (DR features), and OR < 1 and *q* < 0.05 highlights features enriched in non-annotated regions (NR features).

**Fig 1:**
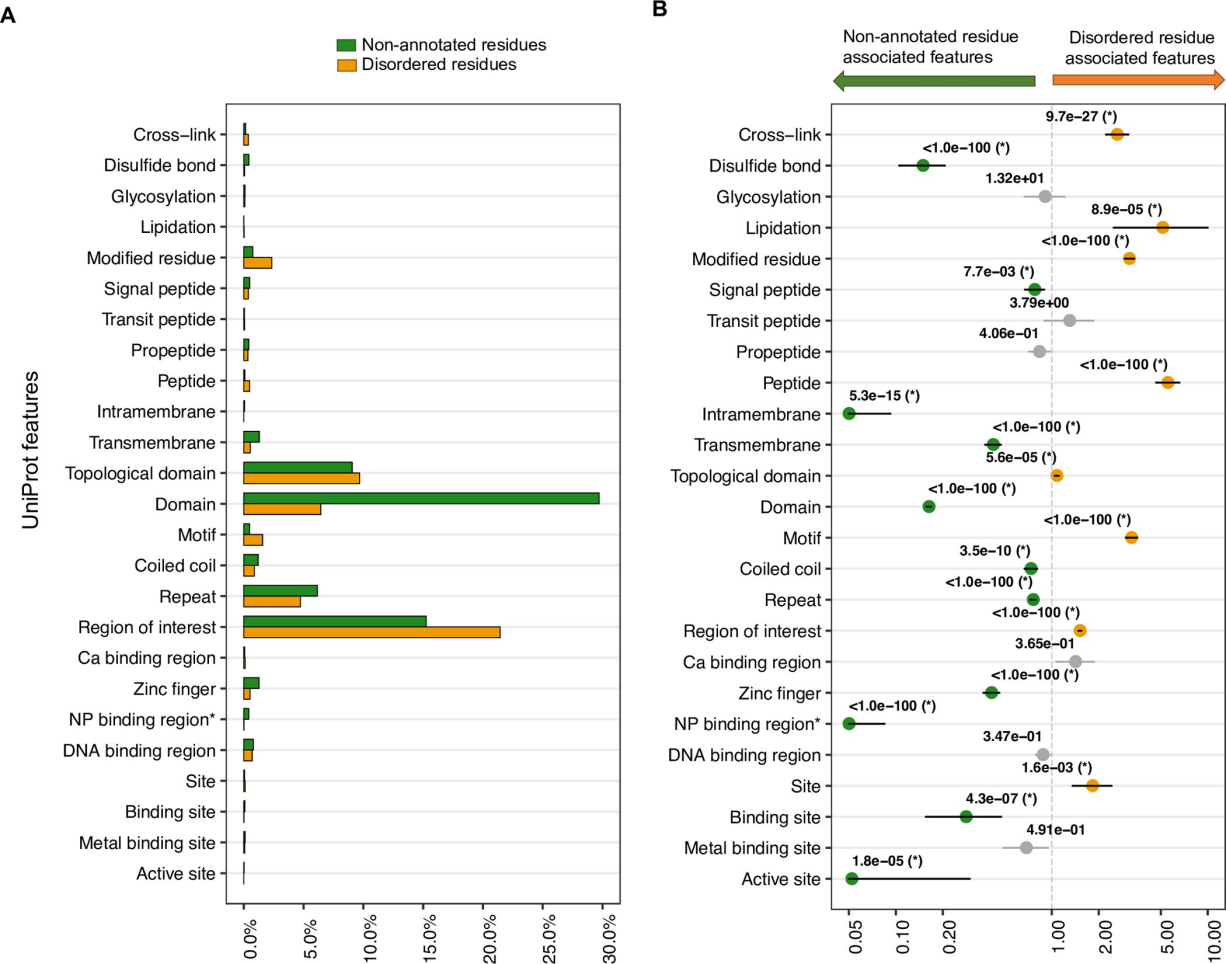
Some UniProt features are more frequent in disordered regions of proteins compared to non-annotated regions. (*A*) Distribution of disordered (total = 58,993) and non-annotated residues (total = 286,113) in 561 IDPs with twenty-five UniProt features. (*B*) Results of statistical association tests (two-tailed Fisher’s Exact test) between UniProt features and disordered residues compared to the non-annotated residues. Circles show the odds ratios (ORs) and are labelled with the corrected p-values (*q*), showing the significance of the association (a value of *q* < 1.0e-100 indicates the maximum significance, see *Materials and Methods*). Horizontal bars show the 95% confidence interval (CI). The OR > 1.0 and OR < 1.0, along with *q* < 0.05, indicate the disordered residue-associated or DR feature (orange circle) and non-annotated residue-associated or NR feature (green circle) feature (y-axis), respectively. The vertical dashed line at OR = 1.0 indicates no association between a residue type (DR or NR) and a feature. To facilitate the visualization, minimum and maximum values of OR along the x-axis are set to 0.05 and 10.0, respectively. Non-significant associations (*q* ≥ 0.05) are indicated by gray CI bars and circles. NP binding region* indicates “Nucleotide phosphate binding region”.

“Regions of interest” (annotated in UniProt to indicate protein regions with experimentally defined roles such as mediating protein-protein interactions or biological processes, regions of multifunctional enzymes or fusion proteins, etc.) in IDPs had the highest fraction of all disordered residues (21.4% of 58,993, **Fig 1A**), whereas “domains” (a feature annotation in UniProt to designate protein segments that represent a specific combination of secondary structures in 3D, organized into a characteristic fold) had the highest proportion of all non-annotated residues (~30% of 286,113, **Fig 1A**). These data contributed to our results identifying firstly “region of interest” as a DR feature (OR = 1.5, *q* < 1.0e-100, **Fig 1B**) and secondly, “domain” as an NR feature (OR = 0.2, *q* < 1.0e-100, **Fig 1B**). Given that IDPs often have a mosaic organization with a hybrid of ordered and disordered domains [18], the aforementioned results may indicate that the non-annotated regions of these IDPs are mostly composed of ordered or structured residues.

A minor fraction (0.2%, 541 out of 345,106) of all residues in 561 IDPs studied here constitute biologically active peptides, i.e. small polypeptides of ≤ 30 amino acids with a well-defined biological activity. Over 53% of these 541 residues are located in disordered regions and the odds of disordered residues to be part of such peptides is 5.5 times higher than that for the non-annotated residues (*q* < 1.0e-100, **Fig 1B**). Altogether, 8 out of 25 features had a higher burden in IDRs (DR features, see description of all UniProt features in *Materials and Methods*: “Collection of UniProt features”) including lipidation sites (OR = 5.1, *q* = 8.5e-05), motifs (OR = 3.2, *q* < 1.0e-100), modified residues (OR = 3.1, *q* < 1.0e-100), cross-links (OR = 2.6, *q* = 9.7e-27), sites (OR = 1.8, *q* = 1.6e-03), and topological domain (OR = 1.1, *q* = 5.6e-03). On the other hand, 11 UniProt features were found to be enriched in non-annotated regions (NR features, **Fig 1B**); active sites (OR = 0.05, *q* = 1.8e-05), nucleotide phosphate binding regions (OR = 0.05, *q* < 1.0e-100) and intramembrane regions (OR = 0.05, *q* = 5.3e-15) being the three most prominent NR features. The remaining six UniProt features showed no significant association with either residue type.

### Distribution of UniProt features in different categories of IDRs

**Fig 2:**
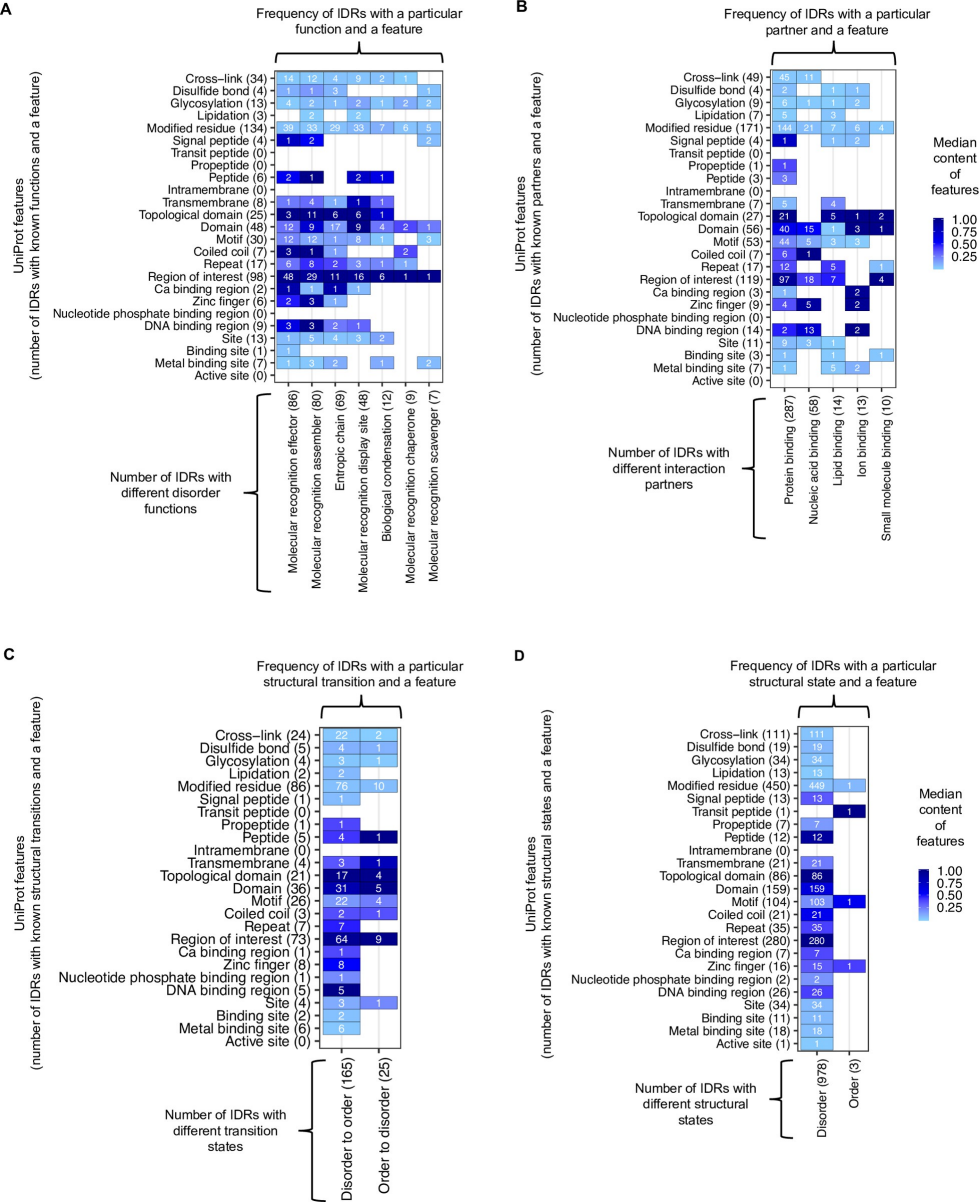
**Median content (the median fraction of residues in IDRs annotated with a feature) of twenty-five UniProt features in different categories of IDRs that** (*A*) perform different functions; (*B*) interact with different partners; (*C*) undergo structural transitions; (*D*) adopt different structural states. In each plot, the x-axis shows the count of IDRs of different categories as annotated in the DisProt database. The y-axis shows the UniProt features (i.e., sites of interest in proteins) and the number of IDRs that have these features. Each cell is labeled with the frequency of IDRs of a specific category (x-axis) and that have a given feature (y-axis). For example, 98 IDRs with known disorder functions overlaps with protein segments annotated as “region of interest”, and 29 of these IDRs are molecular recognition assemblers. Darker color indicates that a higher fraction of residues in IDRs is annotated with the corresponding feature (i.e. the median content of the feature).

Having identified the disordered residue-associated (DR) features (**Fig 1B**), here we looked for any variability in the distribution of UniProt features in different categories of IDRs. Annotations of IDR categories were obtained from the second level of the hierarchy of Disorder Ontology defined in the DisProt database [36], describing the function, interaction partner, structural transition, and structural state of IDRs (see the number of IDRs in different categories in **S2 Table**). The frequency of IDRs with different features and categories along with the median content of each feature, i.e., the median fraction of residues in IDRs annotated with a feature, is shown in **Fig 2**. Additionally, the frequency distributions of residues in IDRs annotated with twenty-five UniProt features are available in **S1**–**S4 Figs**. Note that these frequencies are calculated based on IDRs that are annotated with a category in the DisProt database, and strikingly, only a modest fraction of all IDRs (total = 981) are annotated with functions (28%), interaction partners (37%) and structural transitions (19%), highlighting that current knowledge about the functional roles of IDP is fairly limited.

Two DR features, “region of interest” and “modified residues” (i.e. sites that undergo various post-translational modifications), were present in IDRs of all functions (**Fig 2A**). “Regions of interest” mostly found to overlap with the entire length of IDRs (median content of this feature > 80%, **Fig 2**). About 49% of 98 IDRs annotated with “regions of interest” and with a known function (**Fig 2A**), are molecular recognition effectors that are known to modulate the activity of partner molecules like inhibitors, activators, etc. At the same time, 82% of 119 IDRs annotated with “regions of interest” and with a known interaction partner, bind to another protein molecule (“protein binding”, **Fig 2B**). Two other UniProt features that we found to be common in IDRs are: motifs (≤ 20 residues long sequence motifs of biological interest) and cross-links, i.e., residues participating in covalent linkage(s) between proteins including ubiquitin conjugation, SUMOylation, transglutamination, thioether and thioester bonds. IDRs containing motifs and cross-links are predominantly effectors and assemblers (**Fig 2A**). Another DR feature resulting from our analysis is “sites” which specify cleavage sites, inhibitory sites, etc. As an example, an IDR in the Amyloid-beta precursor protein contains 9 cleavage sites and is annotated in the “biological condensation” disorder function category (**S1 Fig**).

Alongside DR features, we also noticed several non-annotated residue-associated (NR) features (**Fig 1B**) to be frequently present in IDRs: “domain” is the most striking one and is located in all categories of IDRs (**Fig 2**). A puzzling observation was that about 35% (17 out of 48) of all IDRs that overlap with “domains” are “entropic chains”, which are defined as carrying out functions directly enabled by their conformational disorder (**Fig 2A**). Moreover, three short entropic chain IDRs (< 30 residues long) had cysteine residues participating in disulfide bonds (**Fig 2A**), which are *in general* depleted in IDRs (**Fig 1B**). Similarly, zinc fingers, which are primarily located in non-annotated regions of IDPs (**Fig 1B**), are present in 9 IDRs with annotated interaction partners (**Fig 2B**); of these, five are nucleic acid binding IDRs. Assuming that the non-annotated regions in IDPs represent mostly ordered, or at least not entirely disordered regions, these results seem to indicate that there is no well-defined boundary between the functional space of the ordered and disordered regions of IDPs.

### Prevalence of population and pathogenic genetic variations in IDPs

Given that IDPs/IDRs are abundant in nature [42,43] and also carry many features important for protein function (**Figs 1** and **2**, “UniProt features”), it is timely to systematically evaluate the prevalence of genetic variants of IDPs in the general population as well as in patients, and contrast the putative features of IDRs that are most perturbed by disease-associated “pathogenic” mutations with those affected by tolerated “population” mutations.

**Fig 3:**
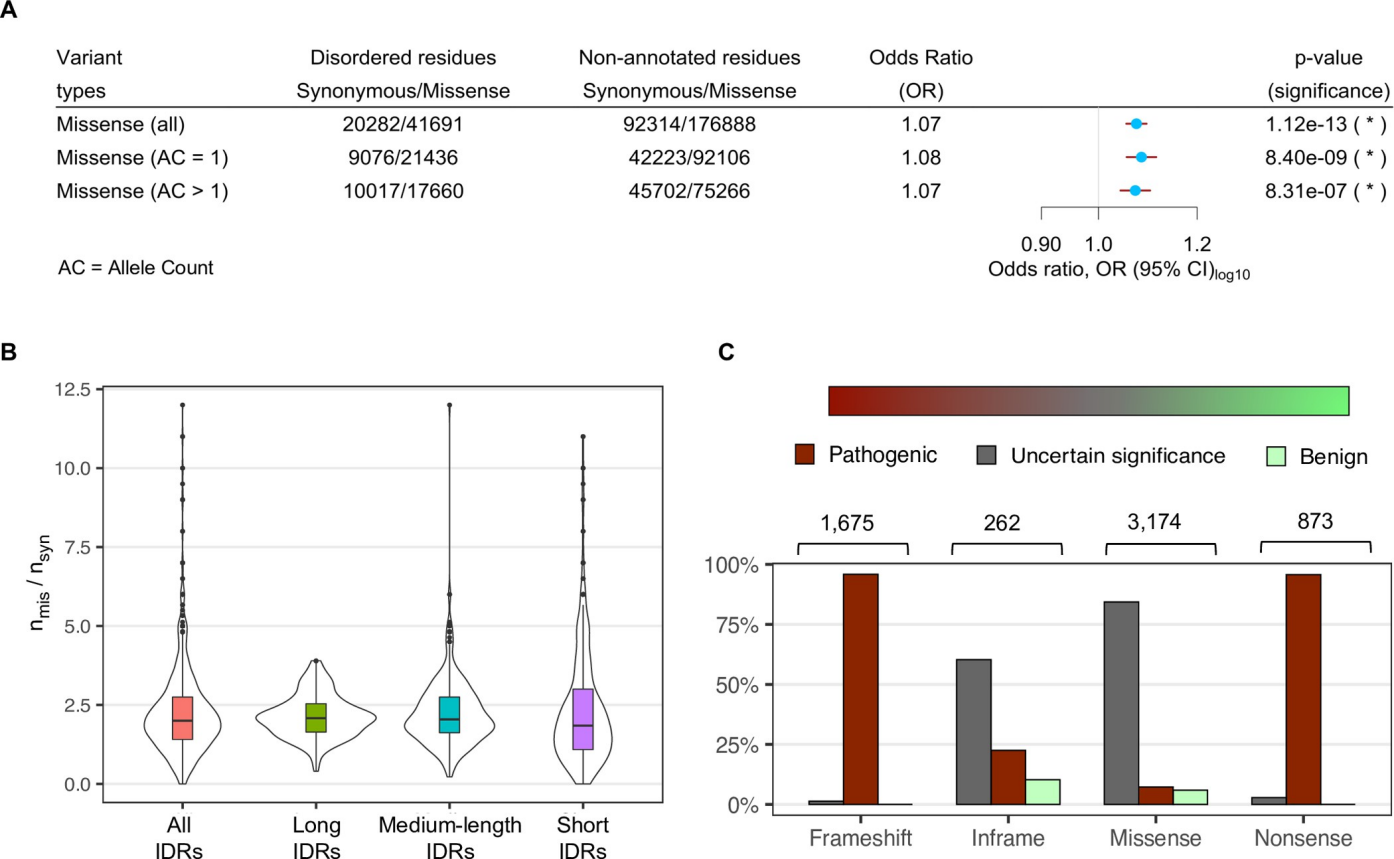
Genetic variations in disordered regions often provide functional advantages to the protein, while for many IDPs, they contribute to the disease phenotype. (*A*) Association of single amino acid-changing missense variations and silent synonymous variations from gnomAD database (a database of variants from relatively healthy individuals) with disordered residues compared to non-annotated residues of 548 human IDPs, calculated using the two-tailed Fisher’s Exact test. Missense variations are marginally (OR = 1.1) but significantly (indicated by “*”) enriched in disordered regions, considering all variants together, and also separately in rare (allele count, AC = 1) and frequent variants (AC > 1). (*B*) Distribution of ratios of missense to synonymous (n_mis_/n_syn_) variations from gnomAD database in all IDRs, and short (≤ 30 residues), medium-length (30 < residues ≤ 100) and long (>100 residues) IDRs. Irrespective of the length, the median of missense-to-synonymous variation ratio in IDRs is over 2.0, showing that point mutations in IDRs can be advantageous. (*C*) Fraction of benign, uncertain significance, and pathogenic mutations of different types (frameshift, inframe, missense and nonsense) observed in patients, affecting disordered regions of 96 human IDPs, collected from ClinVar database.

In order to measure the likelihood of population variations in IDRs, we collected variant data from the genome aggregation database (gnomAD) [40], which represents variants observed in healthy individuals. From gnomAD, we obtained 41,691 and 176,888 missense mutations (an amino acid change caused by a single base substitution) in IDRs and non-annotated regions, respectively, of 548 IDPs. These 548 IDPs are a subset of the initially selected 561 IDPs in our dataset for which variant data were available (*Materials and Methods* for variant collection steps and **S3 Table** for variant counts). Similarly, we collected 20,282 and 92,314 synonymous mutations (no change in protein sequence following a single base substitution) located in IDRs and non-annotated regions, respectively, of the same 548 IDPs. We then measured the association of amino acid acid-changing missense and silent synonymous variations with the reference residue type (disordered and non-annotated residues). The odds ratio (or OR) of missense variations for disordered residues compared to non-annotated residues is 1.1 (p = 1.12e-13, **Fig 3A**), meaning that the odds of observing a missense mutation over a synonymous one on a residue, increases about 10% when that residue is disordered. Additionally, we performed the same analysis separately for singleton (allele count, AC = 1) and multiton (AC > 1) variants. We obtained similar results for both rare (AC = 1) and relatively frequent variants (AC > 1), that is, disordered residues sustain a slight but significant burden of missense variations compared to synonymous changes in the general population (**Fig 3A**).

To further investigate the rate of amino acid change in the disordered regions, we computed region-wise ratio of missense to synonymous variations (n_mis_/n_syn_) observed in the general population for each of the 548 IDRs (**Fig 3B**). The ratios were separately calculated for all IDRs (count = 945), and then also for short (length ≤ 30 residues, IDR count = 450), medium-length (30 < residues ≤ 100, IDR count = 306), and long (>100 residues, IDR count = 189) IDRs. On average, the n_mis_/n_syn_ for all IDRs was 2.3, with a maximum of 12.0, suggesting that amino acid substitutions in IDRs may be advantageous or neutral. Overall, a higher fraction of all population missense variations was observed in long IDRs (59%) than in medium-length and short IDRs (29% and 12%, respectively). Moreover, the median n_mis_/n_syn_ for long and medium-length IDRs were slightly higher (~2.0) than that of short IDRs (~1.8). However, surprisingly, we also identified 39 very short, disordered regions (< 20 residues) that carried over 5 times more missense than synonymous mutations (n_miss_/n_syn_ ≥ 5.0, **Fig 3B**, **S4 Table**).

In addition to identifying the prevalence of “population” variations in IDRs, we also investigated the presence of “pathogenic” mutations in these regions, as available in the database of clinically identified and interpreted germline variants called ClinVar [41]. We collected four types of protein changing variations: missense, nonsense, frameshift and inframe (see *Materials and Methods* for variant collection steps and **S3 Table** for variant counts), with different level of clinical significance such as benign or likely-benign (jointly referred to as benign), pathogenic or likely-pathogenic (jointly referred to as pathogenic) and variants of uncertain clinical significance (VUS). In total, we gathered 5,830 variations located in experimentally annotated disordered regions of 96 IDPs (out of 561 IDPs studied in this work, *Materials and Methods*), which amount to about 37% of all clinically found variations in these 96 proteins Introduction of a premature stop codon by a nonsense mutation and a framing error caused by a frameshift insertion or deletion mutation, commonly lead to either the complete absence of the protein or an altered and/or truncated copy thereof; over 95% of all such mutations hitting an IDR has been found to be pathogenic (**Fig 3C**). About 84% and 60% of all missense (single amino acid substitution) and inframe (a few amino acid change) variations affecting IDRs instead are of uncertain significance, meaning that the pathogenicity of these mutations and their implication in the disease phenotype cannot be established with the available set of evidences [44].

### Identification of IDRs ‘intolerant’ to mutation

**Fig 4:**
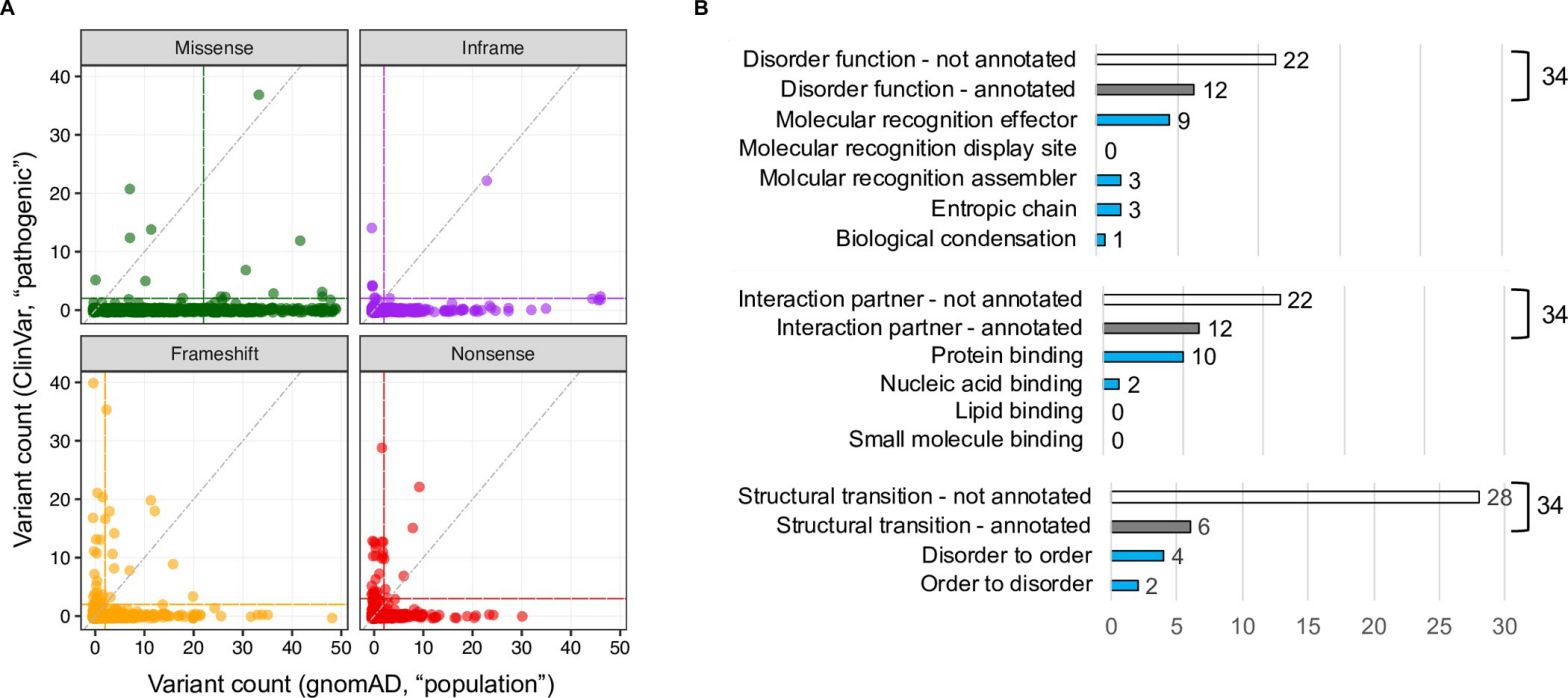
Identification of mutation-intolerant IDRs and their categories. (*A*) Number of population variants from gnomAD (x-axis) compared to the number of pathogenic variants from ClinVar (y-axis) for each IDR. The scatter plots are generated separately for four different types of mutations. The vertical and horizontal lines in each plot show the median count of population and pathogenic variants of a type from all IDRs. Circles located to the left of the vertical line, above both the horizontal and diagonal lines correspond to the IDRs “intolerant” to a particular type of mutation (mutation-intolerant IDRs). Circles located to the right to the vertical line, and below both the horizontal and diagonal lines correspond to the IDRs “tolerant” to a particular type of mutation (mutation-tolerant IDRs) In total, we identified 34 and 533 IDRs that are intolerant and tolerant to different types of mutation, respectively. (*B*) Number of mutation-intolerant IDRs (total = 34) of different categories in terms of disorder functions, interaction partners and structural transitions. Hollow and solid (gray) bars correspond to total mutation-intolerant IDRs that are not-annotated and annotated (with a category), respectively. Blue-colored solid bars represent mutation-intolerant IDRs with known function, interaction partners and structural transitions.

As shown in **Fig 3A and 3B**, many IDRs are robust to amino acid substitutions. However, at the same time, a considerable number of amino acid-changing variations affecting IDRs are pathogenic (**Fig 3C**). It would thus be instructive to identify the IDRs in which different types of protein-altering mutations are absent or kept at low frequency in large population samples and that, at the same time, are commonly present in patients; these would be IDRs that are intolerant to mutations. To find such “mutation-intolerant” disorder regions, we compared the frequency of population and pathogenic mutations from gnomAD [40]) and ClinVar databases [41], respectively, in each IDR. It is expected that the frequency of different types of mutations will be different across the general population and patients. For example, early termination or slippage of the reading frame by nonsense and frameshift mutations is likely to be more severe, and therefore less frequent in the general population, compared to point mutations or an inframe deletion or insertion event. To account for this variability, we searched for mutation-intolerant IDRs independently for four different types of mutation (missense, inframe, frameshift and nonsense, **Fig 4A**). First, we derived the median frequency counts at which these four types of pathogenic and population mutations occur in IDRs. Then taking these median values as expected frequency, we searched for exceptions: that is, IDRs having more than expected pathogenic mutations as well as less than expected population variations of a specific type owing to intolerance to that type of mutation (**Fig 4A**). Simply stated, we defined an IDR as intolerant to a type of mutation if it meets all of the following criteria: it had: (*i*) a higher number of pathogenic than population mutations; (*ii*) a higher number of pathogenic mutations than the expectation (i.e. the median frequency of pathogenic mutations observed in all IDRs); (*iii*) a lower (or equal) number of population mutations compared to the expectation (i.e. median frequency of population mutations observed in all IDRs). Similarly, we identified disordered regions that comply with the opposite of all three conditions listed above; those IDRs are referred to as tolerant to mutations or mutation-tolerant (*Materials and Methods* and **S4 Table** for the list of mutation-intolerant and tolerant IDRs).

In total, we catalogued 34 disordered regions that are intolerant to different types of mutations (**Table 1**); 11 of them are short (≤ 30 residues) and 7 are long (>100 residues) IDRs. Most of the identified “mutation-intolerant” IDRs were intolerant to frameshift (71%, n = 24) and nonsense (62%, n = 21) mutations, while 4 and 7 IDRs were found intolerant to missense and inframe mutations, respectively. The IDR that had the highest number of frameshift (n = 77) and nonsense (n = 13) variations out of all 34 mutation-intolerant IDRs (**Table 1** and **S5A Fig**), is located in the Methyl-CpG-binding protein 2 encoded by gene *MECP2* (DisProt ID: DP00539r004, a molecular recognition effector/inhibitor that binds with the methylated DNA). *MECP2* variants in this IDR are associated with several neurodevelopmental and psychiatric disorders [41,45], e.g., severe neonatal-onset encephalopathy with microcephaly, Rett syndrome, focal epilepsy, intellectual disability, and autism (**S5 Table**). Strikingly, this particular disordered region (residue: 207–310, **Table 1**) in MECP2 protein never acquires a premature stop codon in the general population (**Table 1**), suggesting that single nucleotide variations (SNVs) in this region leading to the termination of the protein should be under extreme purifying selection [46].

**Table 1:** Intrinsically disordered regions (IDRs) that are intolerant to mutations and their categories.

Protein	IDR (start)	IDR (end)	IDR (length)	Disorder function	Interaction partner	Structural transition	Variant count (ClinVar / gnomAD)			
							frameshift	nonsense	missense	inframe
BMPR1A	24	54	31	-	-	-	5 / 0*	1 / 0	0 / 12	2 / 0*
BRCA2	21	39	19	-	Protein binding	Disorder to order	11 / 0*	13 / 2	1 / 24	2 / 0*
CDKN1B	1	198	198	Inhibitor	Protein binding	-	8 / 7	7 / 1*	1 / 179	0 / 2
CDKN1B	25	90	66	Inhibitor	Protein binding	Disorder to order	4 / 2	5 / 0*	1 / 52	0 / 1
CDKN1B	55	95	41	-	Protein binding	-	3 / 1*	4 / 0*	1 / 52	0 / 1
CDKN1C	27	97	71	Molecular recognition effector	Protein binding	-	2 / 0*	1 / 4	0 / 68	0 / 0
CDKN2A	1	37	37	-	-	-	1 / 4	3 / 0	0 / 62	0 / 0
COL7A1	1940	1978	39	-	-	-	2 / 0*	1 / 0	1 / 23	0 / 0
CSTB	1	67	67	Prion	Protein binding	-	1 / 1	3 / 1*	3 / 46	0 / 1
DDX3X	1	167	167	-	-	-	7 / 0*	6 / 0*	5 / 0*	0 / 0
EMD	1	187	187	-	-	-	11 / 0*	10 / 0*	5 / 76	2 / 3
EMD	67	170	104	-	-	-	4 / 0*	4 / 0*	0 / 50	2 / 1*
KIT	544	565	22	-	-	-	1 / 0	3 / 1	21 / 7	14 / 0
LDLR	163	175	13	Flexible linker/spacer; Tethering	-	-	3 / 0*	5 / 2	14 / 11*	1 / 0
LDLR	354	393	40	-	-	-	13 / 1*	10 / 2	37 / 33	4 / 0*
MECP2	1	75	75	Molecular recognition assembler	Nucleic acid binding	-	21 / 0*	10 / 0*	2 / 26	0 / 1
MECP2	165	210	46	Assembler	-	-	13 / 0*	13 / 0*	2 / 46	1 / 1
MECP2	207	310	104	Inhibitor	Nucleic acid binding		77 / 0*	13 / 0*	14 / 60	4 / 0*
MECP2	261	330	70	-	-	-	40 / 0*	4 / 0*	12 / 42	4 / 0*
NFKBIA	1	66	66	-	-	-	0 / 0	4 / 1*	7 / 31	0 / 2
PAX6	1	130	130	-	-	Disorder to order	18 / 3	12 / 0*	23 / 69	0 / 0
RAF1	233	259	27	-	-	-	0 / 0	0 / 0	12 / 7*	0 / 0
RB1	245	269	25	-	-	-	4 / 0*	2 / 0	0 / 15	0 / 0
RB1	346	370	25	Flexible linker/spacer	Protein binding	Order to disorder	3 / 0*	1 / 0	0 / 11	0 / 1
RB1	355	370	16	-	-	-	2 / 0*	1 / 0	0 / 8	0 / 1
RB1	500	511	12	-	-	-	2 / 0*	1 / 0	0 / 1	0 / 0
RB1	500	513	14	-	-	-	2 / 1*	1 / 0	0 / 2	0 / 0
RB1	577	615	39	Flexible linker/spacer	Protein binding	-	2 / 0*	4 / 0*	0 / 22	0 / 0
RB1	786	928	143	-	-	-	6 / 0*	12 / 0*	1 / 65	0 / 1
SMAD4	297	306	10	-	Protein binding	Order to disorder	1 / 0	3 / 0*	0 / 4	0 / 0
SUFU	279	360	82	-	-	-	2 / 1*	1 / 0	0 / 58	0 / 0
TP53	60	92	33	Activator	-	-	17 / 0*	4 / 0*	0 / 36	0 / 2
TP53	291	312	22	-	-	-	8 / 4	4 / 0*	2 / 26	1 / 0
WAS	201	268	68	Molecular recognition effector	Protein binding	Disorder to order	2 / 0*	3 / 1*	1 / 17	0 / 1

* The corresponding IDR is intolerant of that type of mutation

- IDR category annotation is not available

It is important to note that most of the mutation-intolerant IDRs identified here are not annotated with any function or interaction partner in the DisProt database (**Fig 4B** and **Table 1**). Only 12 IDRs have experimentally verified disorder functions; 9 of these contribute to molecular recognition: 6 effectors (modulate partners’ activity) and 3 assemblers (participate in or facilitate the assembly of complexes). Three relatively short (19 to 39 residues long) entropic chain IDRs were also found to be intolerant to mutations; all of them are flexible linkers or spacers, that allow movement between adjacent binding elements or domains in the protein. One notable example is that of Retinoblastoma protein (RB1, **Table 1**): Multiple frameshift and stop-gained variants of RB1 with two such altered linkers/spacers are implicated in a very rare retinoblastoma condition (**S5 Table**). Additionally, 10 mutation-intolerant IDRs were annotated with experimentally determined interaction partners in the DisProt database (“protein binding”, **Fig 4B**). One of these is an effector region located in the *WAS* encoded protein (WASp) that regulates actin filament reorganization and polymerization [47] and are also annotated as undergoing a disordered to order transition. SNVs in this disordered region leading to the truncation of the protein is associated with severe congenital neutropenia and rare Wiskott-Aldrich syndrome (**S5 Table**).

### Characterization of IDRs that are intolerant to mutations

Having stratified the mutation-intolerant IDRs that are predominantly affected by disease-associated variants (**Fig 4** and **Table 1**), next we quantified the enrichment of 25 UniProt features in these mutation-intolerant IDRs as explained previously, compared to mutation-tolerant IDRs.

**Fig 5:**
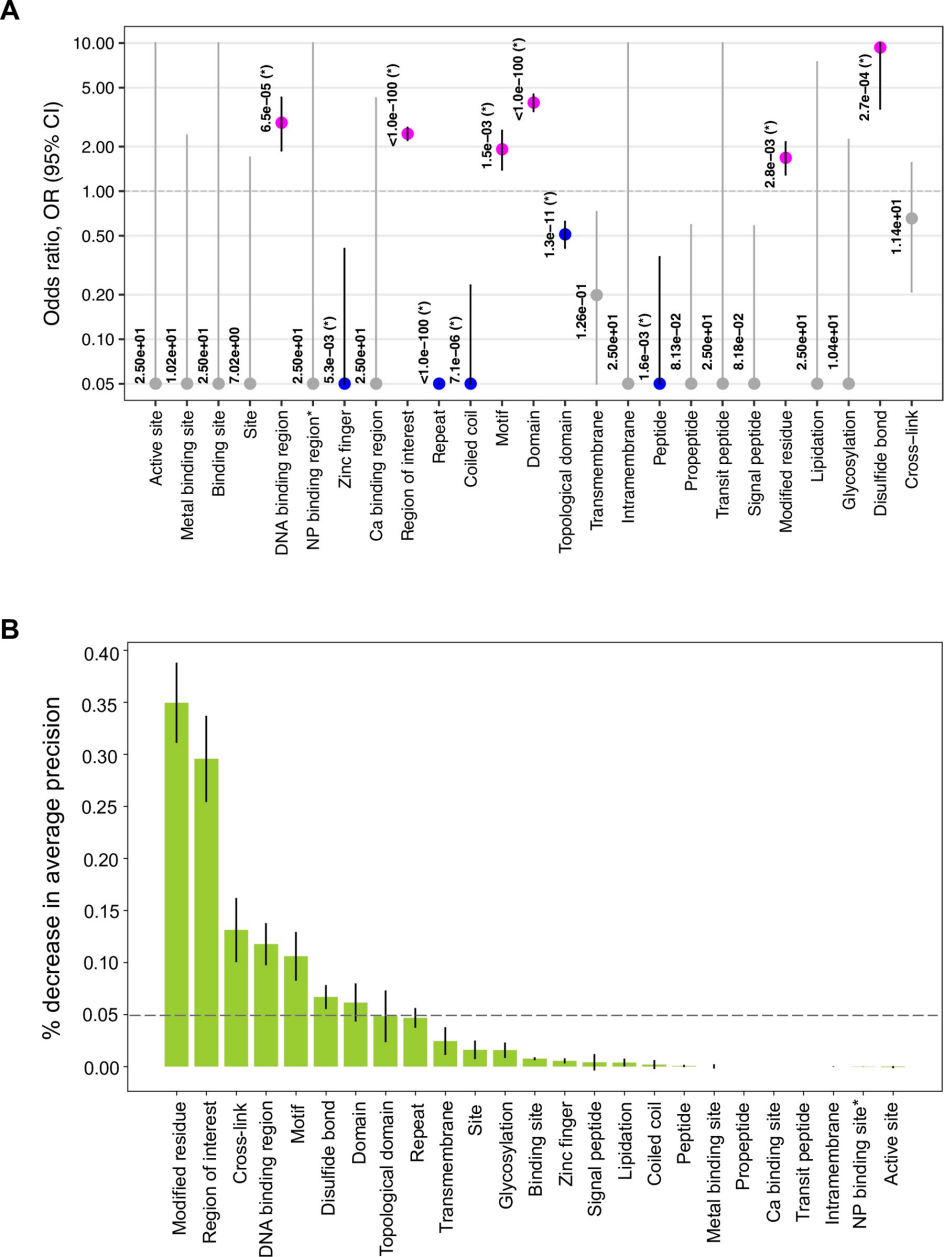
Mutation-intolerant IDRs carry characteristic UniProt features compared to relatively tolerant IDRs. (*A*) Results of association (two-tailed Fisher’s Exact test) between different UniProt features (x-axis) and residues of mutation-intolerant and tolerant IDRs (Fig 4A). Circles show the odds ratios (ORs) and are labelled with the corrected p-values (*q*), showing the significance of the association (a value of *q* < 1.0e-100 indicates the maximum significance, see *Materials and Methods*). Vertical bars show the 95% confidence interval (CI). OR > 1.0 and OR < 1.0, along with *q* < 0.05, indicate that the feature is associated with the mutation-intolerant (magenta circle) and mutation-tolerant (blue circle) IDRs, respectively. The horizontal dashed line at OR = 1.0 indicates no association between a residue type and a feature. To facilitate the visualization, minimum and maximum values of OR along the y-axis are set to 0.05 and 10.0, respectively. For non-significant associations (*q* ≥ 0.05), CI bars and circles are grey. (*B*) Relative importance of different UniProt features in stratifying mutation-intolerant and tolerant IDRs using a random forest classifier (*Materials and Methods*). The y-axis shows the drop in the average precision of the classifier’s performance when a particular feature (x-axis) is randomly permutated, that is, when the feature’s values are randomly shuffled thereby breaking the relationship between the feature and the true outcome. For example, when the frequency of “modified residues” in IDRs is permutated, the average precision of the classification model decreased by about 35%. This procedure allows us to rank the features according to their importance for accurately predicting mutation-intolerant versus tolerant IDRs. In both panels, NP binding region* indicates “Nucleotide phosphate binding region”.

Six out of 25 UniProt features showed significant association with residues of mutation-intolerant IDRs (**Fig 5A**); interestingly, only three of these features overlapped with the eight features that were found enriched in IDRs *in general* (**Fig 1B**). These three features are: “regions of interest” (OR = 2.4, *q* < 1.0e-100, **Fig 5A**) in proteins with a tendency to be involved in interactions with many partner proteins, short sequence “motifs” (OR = 2.0, *q* < 1.0e-100, **Fig 5A**) that often act as molecular switches and regulate low affinity interactions, and “modified residues” (OR = 1.7, *q* = 2.8e-03), i.e. sites that undergo different post-translation modification (PTM) such as phosphorylation, methylations, acetylation. Surprisingly, disulfide bonds formed between two cysteins from two different proteins or within the same protein chain had 9-fold enrichment in mutation-intolerant IDRs (*q* = 2.7e-04). This feature, and “domains” are *in genera*l depleted in disordered regions (**Fig 1B**), which, however, when present in IDRs and perturbed by mutations, seem to contribute to disease mechanisms. Further, “DNA binding region” showed no significant association with any residue type in IDPs (**Fig 1B**) but was identified as a characteristic feature of mutation-intolerant disordered regions (3-fold, *q* = 6.5e-05, **Fig 5A**).

To further assess how useful the identified features can be for the blind prediction of IDRs that are intolerant to mutations, we quantified the relative importance of UniProt features in the classification of mutation-intolerant versus tolerant IDRs using “permutation feature importance” method [48]. This is a model-agnostic technique to measures the importance of a feature by calculating the decrease in the classifier model’s prediction score after permuting the feature, which breaks the relationship between the feature and the true outcome. A feature is “important” if shuffling its values decreases the model prediction score, because in this case the model relied on the feature for the prediction. A feature is “unimportant” if shuffling its values leaves the model’s score unchanged, because in this case the model ignored the feature for the prediction. For our analysis, we calculated the frequency of all 25 UniProt features in each IDR (e.g., how many “regions of interest” or “motifs” are overlapped with the location of an IDR, **S6 Table**), and fed these frequency counts into the “permutation feature importance” method to estimate their relative importance to stratify mutation-intolerant and mutation-tolerant IDRs. We used Random Forest as the classifier model [49] and “average precision” as the prediction score (**S7 Table** and *Materials and Methods*: “Measuring Relative Feature Importance”). Average precision summarizes the precision-recall curve, commonly used for evaluating the performance of binary classifiers. The output of this analysis was the difference in the precision of the classifier model before and after permutation or shuffling of each feature values (% decrease in average precision, **Fig 5B**).

**Fig 6:**
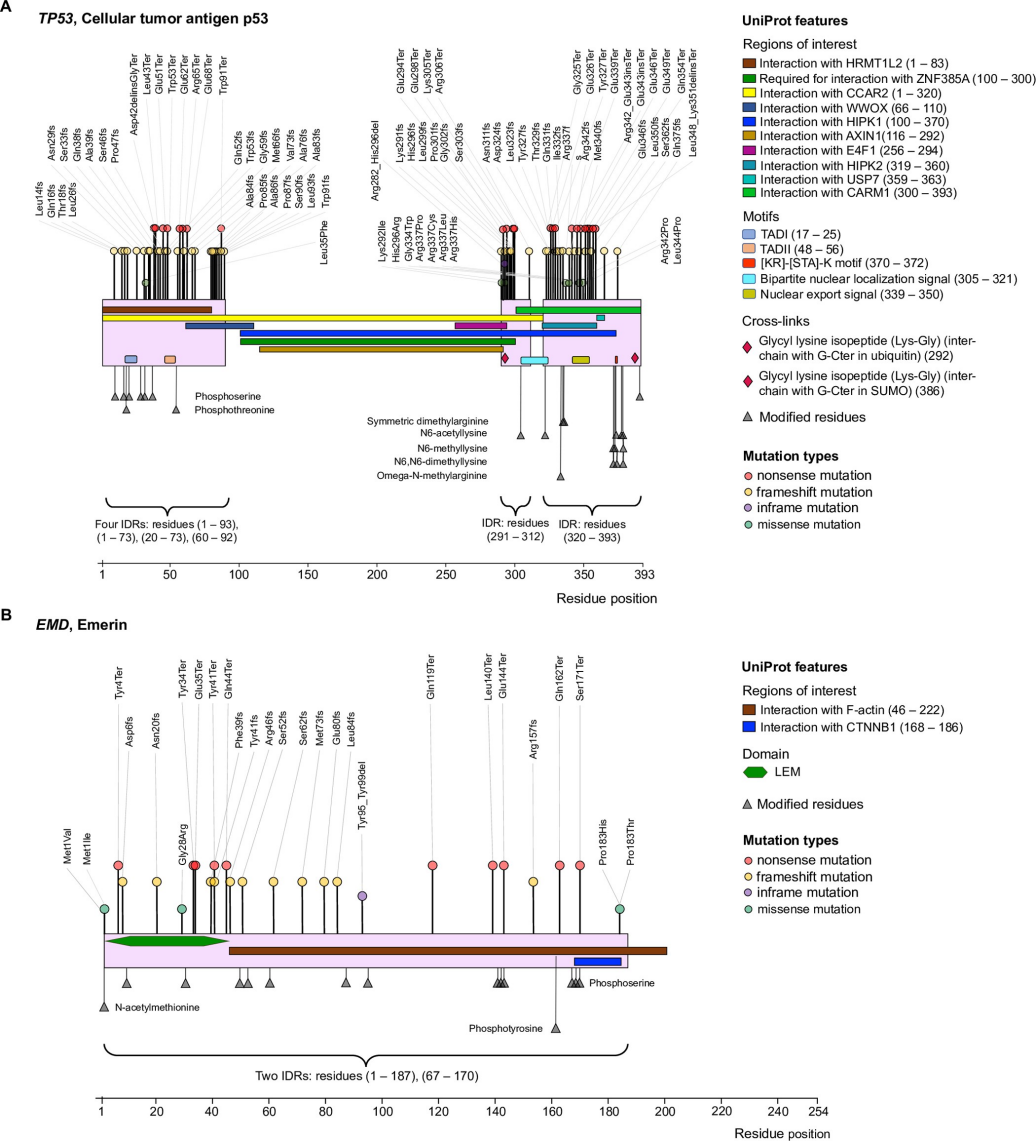
Illustration of mutation-intolerant IDRs with their characteristic UniProt features for two intrinsically disordered proteins. (*A*) The cellular tumor antigen protein p53 has six disordered regions; two of them were identified as intolerant to frameshift and—nonsense mutations: residues 60–92 (an effector IDR) and residues 291–312 (*not annotated* with any IDR category, Table 1). These IDRs contain “regions of interest” that are involved in interactions with many partners (e.g. HRMT1L2, ZNF385A, CCAR2, WWOX, HIPK1, AXIN1, E4F1, HIPK2, USP7, CARM1), have several motifs (TADI, TADII, nuclear localization signal, [KR]-[STA]-K motif, etc.), perform interchain cross-links, and contain over 20 post-translational modification (PTM) sites or “modified residues”. All these UniProt features are identified as the characteristic features of mutation-intolerant IDR, in this study (Fig 5). Mutations affecting these IDRs (partly or in full) in p53 are primarily associated with hereditary cancer-predisposing syndrome, Li-Fraumeni syndrome, ovarian neoplasms, and familial breast cancer. (*B*) Emerin protein has two IDRs and both these IDRs were identified as mutation-intolerant (Table 1), carrying characteristic UniProt features such as F-actin and CTNNB1 binding “region of interest”, LEM domain, and 15 PTM sites (“modified residues”). Pathogenic variations in these IDRs in Emerin are associated with Emery-Dreifuss muscular dystrophy, flexion contracture, muscular diseases, cardiomyopathy, etc. (S5 Table).

Seven out of 25 UniProt features were identified as important features for the classification of mutation-intolerant IDRs from mutation-tolerant IDRs, i.e., permutation of these features resulted in a decrease in the average precision of the classifier’s performance by at least 5% (**Fig 5B**). Particularly, the presence of “modified residue” (PTMs) and “regions of interest” were ranked as the two most influential functional elements of disordered regions that are intolerant of disease-causing germline mutations, with an average loss of precision of about 30% and higher upon permutation (**Fig 5B**). Altogether, six out of seven of these features identified as important features of mutation-intolerant IDRs by the permutation importance method (**Fig 5B**) were also found as significantly enriched in mutation-intolerant IDRs compared to tolerant regions (**Fig 5A**). Concordance between the output of two different approaches applied in this study, OR enrichment analysis and machine learning (**Fig 5A and 5B**), to find the characteristic features of mutation-intolerant IDRs, validates the soundness of our findings. Some notable cases of mutation-intolerant disordered regions with identified features are presented in **Figs 6 and S5**.

## Discussion

The ever-increasing number of experimentally validated disordered regions (IDRs) in different proteomes and their remarkable functional diversity have led to a rapidly growing appreciation of the intrinsic disorder phenomenon [50,51]. Subsequently, several resources have been developed to aggregate both experimental and computationally predicted information on disordered regions in proteins [36–38,52,53]: The Database of Protein Disorder (DisProt) is one of these resources, primarily reporting on experimentally characterized IDRs and their biological function, when known [36]. Strikingly, over 70% of all disordered regions of 561 human IDPs that we collected from DisProt and studied in this work, currently have no function annotation, which limits the understanding of how these proteins function or malfunction when perturbed by genetic mutations.

Sequences of IDPs and IDRs have distinct compositional properties and biases which have been the basis of many computational studies for identification, characterization and prediction of IDRs and their functions [18,27,50]. Disordered regions are enriched with charged and polar amino acids as well as depleted in bulky hydrophobic residues [5,6], leading to a weakened hydrophobic effect, which is usually the main drive for the folding of polypeptides into their compact tertiary structure (natively folded state). In this study, using the annotation of protein sequences with biologically interesting sites and regions from UniProt [28], we identified eight “UniProt features” that are statistically enriched in IDRs (**Fig 1**). Our results recapitulate some of the commonly known functions of IDRs, e.g. the abundance of short linear motifs and PTM sites (“modified residues,” “cross-links,” “lipidations,” etc., **Figs 1** and **S1–S4**), consistently with IDRs’ role in cell signaling and molecular regulation [17,19,54]. Further characterization of the UniProt feature “motif” was performed using the Eukaryotic Linear Motif (ELM) resource [55,56], that organizes experimentally validated short linear motifs into types based on their functions. This showed that 41% and 18% of the motifs overlapping with IDRs in our dataset are ligand sites (LIG), which mediate binding of the ligand protein to its interaction partner, and subcellular targeting sites (TRG), respectively (**S6A Fig** and **S8 Table**). Moreover, by investigating the Gene Ontology terms (GO version 2021-11-20 [57]) for the motifs available in the ELM resource, we found that 53% of these motifs in IDRs are involved in different biological processes such as DNA repair/replication/damage and cell division/death (**S6B Fig**), in agreement with previous studies showing the link between IDPs and these biological processes [9,58,59].

Although the UniProt feature “domain” was not statistically enriched in disordered regions (Fig 1B), we observed an intriguing overlap between these “domains” and experimentally verified IDRs (**Figs 2** and **S1–S4**). Delving deeper into these data, we found that many of the UniProt-annotated “domains” such as the kinase inducible domain (KID), BH3 domain, the Wasp homology domain, overlap with disordered regions of proteins, suggesting that they are intrinsically disordered domains (IDDs) [60–62]. IDDs represent protein regions that conform to the typical definition of domains, i.e. functional, structured and conserved units in their native context, but with the important difference that they are disordered in isolation and only form structure in certain conditions, such as in coupled folding and binding (to a macromolecular partner), upon formation of disulfide bonds, or ion coordination [61,63,64]. To annotate protein regions as “domains”, UniProt uses InterPro resource [65], which employs MobiDB-lite [66] to determine disordered regions; this might be the reason behind UniProt reporting IDDs as “domain”. In our dataset, 105 intrinsically disordered proteins (IDPs) have 124 UniProt-annotated “domains” that overlap with disordered regions (IDRs) of these proteins (**S9 Table**). According to DisProt [36], 30 out of these 124 “domains” have been categorized as having a “disordered” structural state and undergoing a “disorder to order” structural transition—a signature of some intrinsically disordered domains (**S9 Table**). Furthermore, we checked Mutual Folding Induced by Binding (MFIB) [53] and Disordered Binding Site (DIBS) [52] databases, which are repositories for protein complexes formed exclusively by IDPs (homomers or heteromers), and between IDPs and globular partner proteins, respectively. Eleven UniProt-annotated domains, overlapping with IDRs, were also annotated in these databases as being unstructured in isolation but forming structure upon binding (**S9 Table**).

Disordered regions reportedly are frequent targets for positive selection [33,67] but are also shown to be associated with many human diseases when mutated [68–70]. Our results from the analysis of variants from relatively healthy individuals in the general population [40], support the concept underlying the former observation, that is owing to the lack of structural constraints, IDRs evolve relatively fast yet are usually able to preserve their function [31]. About 53% of all IDRs (500 out of 945) in our dataset had a 2 to 12 times higher fraction of missense than synonymous mutations (n_mis_/n_syn_) in the general population (**Fig 3B** and **S4 Table**), suggesting that the substitution of residues in these disordered regions are likely favorable to maintain a large, evolutionarily advantageous basin of diversity in humans [30,31,67]. Interestingly, we noticed that the n_mis_/n_syn_ is consistently greater than or equal to 2 for long IDRs (>100 residues), while that for relatively short IDRs varies widely (**S7 Fig**). We identified 47 and 38 short disordered regions (≤ 30 residues) with n_mis_/n_syn_ ≥ 5.0 and ≤ 0.5, respectively, in two mutually exclusive set of 38 and 29 proteins (**S7 Fig** and **S4 Table**). These results shows that IDRs, particularly short ones, display a remarkable degree of variability in their tolerance to amino acid substitutions.

A method to detect protein regions that are intolerant to a certain type of mutation is to compare the frequency of pathogenic and population mutations of that type in the same region. A “mutation-intolerant” region would harbour higher than expected pathogenic mutations as well as lower than expected population mutations. Applying this method to our set of disordered region, with pathogenic variations collected from ClinVar database [41] and population variations collected from gnomAD database [40], we identified 34 IDRs in 20 proteins that are intolerant to different types of mutation (**Fig 4** and **S5 Table**). Specifically, we identified 21, 24, 7 and 4 IDRs that are intolerant to nonsense, frameshift, inframe insertion/deletion and missense mutations, respectively (**Table 1**). Identification of mutation-intolerant disordered regions in this way, using population genetics data is hitherto underexplored for IDPs, albeit important to prioritize IDRs and IDPs that are essential for the organisms fitness for experimental characterization. Indeed, our results identified disordered regions that are intolerant to partial or full deletion by frameshift and protein-truncating mutations in 8 IDPs (DDX3X, KIT, NFKBIA, PAX6, RB1, SMAD4, SUFU, and WAS). According to a recently developed mutational constraint spectrum using a large population sample [40], these eight intrinsically disordered proteins are among those that, out of all human proteins, have been classified to be intolerant to stop-gained and frameshifts mutations.

To the best of our knowledge, no previous studies have statistically assessed the features of mutation-intolerant compared to mutation-tolerant disordered regions, which could provide insights into the functional elements of IDRs that are particularly vulnerable to trigger pathogenesis when mutated. Thanks to functional site and region annotations at the protein sequence level available in UniProt knowledgebase [28], we were able to investigate any variability in the statistical burden of different functional elements (“UniProt features”) across all IDRs and separately across mutation-intolerant disordered regions. Our results captured features that are (*i*) predominantly located in all IDRs as well as in mutation-intolerant IDRs (such as “modified residues” or PTM sites, short linear motifs, “regions of interest,” **Figs 1** and **5**); (*ii*) depleted in disordered regions *in general* but seemingly frequent in mutation-intolerant disordered regions compared to the mutation-tolerant regions. For example, results of our odds ratio enrichment analysis taking all disordered and non-annotated regions of IDPs (**Fig 1B**) suggest that residues involved in covalent disulfide bonds and that are located in “domain” and “DNA binding region”, are not enriched in disordered regions. However, these functional elements were found specifically enriched in mutation-intolerant IDRs (**Fig 5A**). A case when a disordered region carries the identified features associated with mutation-intolerant IDRs (**Fig 5A**), and impairment of those features by mutations lead to the pathogenesis, is that of the emerin protein (EMD, **Fig 6B**). Emerin is an integral protein of the nuclear inner membrane, which contains a 187-residues long IDR spanning a “domain” (LEM), two “regions of interest” interacting with F-actin and Catenin beta-1, and 15 “modified residues” (**Fig 6B**). All these three UniProt features have been found to be the signature of IDRs that are intolerant to mutations in our study (**Fig 5A**), and indeed, germline variants causing deletion of Emerin or introducing small changes in the IDR of Emerin (i.e. by missense and inframe variations, **Fig 6B** and **S5 Table**) have been found to be responsible for X-linked Emery-Dreifuss muscular dystrophy [71,72]. Notably, IDRs in the emerin protein have no experimentally validated function annotation in DisProt database (**Table 1**), however, using genetic variation data we could assign a level of functional importance to these regions—they are intolerant to genetic mutations—and subsequently could characterize these protein regions.

The validity of our results in identifying the distinct set of features of disordered regions that are intolerant to mutations (**Fig 5A**) is further supported by the outcome of a machine learning-based approach that we applied to quantify the importance of these features in classifying mutation-intolerant and tolerant IDRs. Results show that permutation of characteristic features of mutation-intolerant IDRs, such as “regions of interest”, “modified residue”, reduces the performance of a classifier model by 30% in stratifying mutation-intolerant versus tolerant IDRs (**Fig 5B**). It can, therefore, be projected that the identified set of features of mutation-intolerant IDRs in this study will be valuable in developing a predictor model, to classify disordered regions that do not tolerate mutations from those that are robust to changes on a large scale. Future investigation in this direction on a large scale, incorporating high-quality, predicted IDR annotations from MobiDB database [37], and experimentally validated annotations from DisProt [36] and IDEAL [38] databases, will help detect and characterize novel disease-associated disordered regions across the human proteome.

To summarize, in this study, we leveraged the annotation of functionally relevant sites and regions in proteins from UniProt to systematically characterize the disordered and non-annotated regions in human intrinsically disordered proteins. We then extended our analysis using data from human genetic variants to identify IDRs with a relatively high frequency of pathogenic mutations. Our results show that disordered regions that contribute to disease mechanisms upon mutation (mutation-intolerant) carry a characteristic set of functional features compared to the disordered regions that undergo rapid evolution in the general population (mutation-tolerant). By bringing the genetic diversity information into the classification of IDRs that are intolerant to mutations, we propose a new way of annotating functionally important disordered regions: our method will help to select pathogenic variant-enriched disordered regions for functional assay and will aid in generating hypotheses to target the corresponding proteins with therapeutic strategies.

## Materials and methods

### Collection and annotation of disordered regions

Disordered residue and region annotations for 567 human IDPs were collected from the DisProt database (release 2020_06, version 8.0.2) [36], containing 1006 disordered regions (IDRs). Finally, we analyzed 561 proteins with 981 IDRs (**S1 Table**), for which the disorder annotation was available for the canonical protein isoform sequence per UniProt knowledgebase [28]. These 561 proteins are comprised of 58,993 disordered amino acid residues and the rest of the 286,113 residues are referred to as “non-annotated” in this study. Further, we collected the annotations for IDRs, when available, with different disorder functions (seven categories: molecular recognition effector, assembler, display site, scavenger, schaperone, entropic chain, and biological condensation), interaction partners (five categories: protein binding, nucleic acid binding, lipid binding, ion binding, and small molecule binding), transition states (two categories: disorder to order and order to disorder) and structural states (two categories: order and disorder). The number of IDRs of different categories are reported in the **S2 Table**.

### Collection of “UniProt features”

UniProt [36] records sequence annotations describing the regions or sites of interest in proteins (https://www.uniprot.org/help/sequence_annotation). We collected twenty-five different annotations frorm UniProt (release 2020_02) for each amino acid residue, referred to as “UniProt feature” in this study. These features include: active site, metal binding site, binding site (for any chemical group such as co-enzyme, prosthetic group), site (any other interesting amino acid residues, e.g., cleavage sites, inhibitory sites for proteases, breakpoint sites for fusion proteins due to chromosomal rearrangement), DNA binding region, nucleotide phosphate binding region, zinc finger, Ca binding region, region of interest (a region in sequence with an experimentally determined role), repeat, coiled coil, motif, domain, topological domain, transmembrane, intramembrane, peptide (extent of an active peptide in the mature protein), propeptide (part of a protein that is cleaved during maturation or activation), transit peptide, signal peptide, modified residue (excluding lipids, glycans and protein cross-links), lipidation, glycosylation, disulfide bond, cross-links (residues participating in covalent linkage(s) between proteins include ubiquitin conjugation, SUMOylation, transglutamination, thioether bonds and thioester bonds).

### Collection of genetic variants

Genome Aggregation Database (gnomAD) v2.1 containing the variation (i.e., mutation) data from 125,748 exomes and 15,708 genomes of relatively healthy individuals [40] was searched to collect “population” variations. For 548 out of 561 IDPs, 350,044 population variations were obtained. This dataset included four types of protein-changing variations: (i) missense (single amino acid substitution led by single nucleotide change); (*ii*) nonsense (truncation of protein caused by a premature stop codon); (*iii*) frameshift (insertion or deletion causing shifting of the triplet reading frame); (*iv*) inframe (insertion or deletion that does not cause a shift in the reading frame, leading to a few amino acid change), and the synonymous variation (no change in protein upon single nucleotide change) data were collected. The allele count information for each variant were also aggregated to analyze the rare (allele count = 1) and relatively frequent (allele count > 1) population variants separately.

In addition, ClinVar database [41], that records variations (primarily germline) observed in patients and their relationship to human health, was searched to collect disease-associated variations. ClinVar data were available for 96 IDPs, harbouring 21,668 variations. We further collected the phenotype or disease information associated with the variants and their clinical significance (i.e., pathogenic/likely pathogenic, benign/likely benign, uncertain significance, etc.), as determined by the current guidelines proposed by the American College of Medical Genetics and Genomics community [44]. Four types of variations were collected: missense, stop-gained/nonsense, frameshifts, and inframe. Individual counts of variations of different types, obtained from both gnomAD and ClinVar databases, affecting disordered and non-annotated regions of IDPs are reported in **S3 Table**.

### Defining “mutation-intolerant” and “mutation-tolerant” IDRs

For each disordered region (IDR), we computed the frequency counts of pathogenic and population mutations of a type that are located in the region, denoted as $N_{mutation\;type}^{pathogenic}$ and $N_{mutation\;type}^{population}$. Here the *mutation type* can be: missense, nonsense, frameshift and inframe. Using median frequency counts of pathogenic and population mutation of a type observed in all IDRs (${median}_{mutation\;type}^{pathogenic}$ and ${median}_{mutation\;type}^{population}$) as the threshold or expected frequency, we defined an IDR as intolerant to a type mutation if for that IDR the following three conditions are met: (*i*) $N_{mutation\;type}^{pathogenic}>N_{mutation\;type}^{population}$, (*ii*) $N_{mutation\;type}^{pathogenic}>{median}_{mutation\;type}^{pathogenic}$, and (*iii*) $N_{mutation\;type}^{population}$ ≤ ${median}_{mutation\;type}^{population}$. Conversely, an IDR was identified as mutation-tolerant when the opposite three conditions are met: (*i*) $N_{mutation\;type}^{population}>N_{mutation\;type}^{pathogenic}$; (*ii*) $N_{mutation\;type}^{population}>{median}_{mutation\;type}^{population}$; (*iii*) $N_{mutation\;type}^{pathogenic}$ ≤ ${median}_{mutation\;type}^{pathogenic}$. For our set of IDRs, the ${median}_{missense}^{pathogenic},\;{median}_{inframe}^{pathogenic},\;{median}_{frameshift}^{pathogenic}$, and ${median}_{nonsense}^{pathogenic}$ are 2 (**Fig 4A**). And the ${median}_{missense}^{population},\;{median}_{inframe}^{population},\;{median}_{frameshift}^{population}$, and ${median}_{nonsense}^{population}$ are 22, 2, 2, and 2, respectively (**Fig 4A**). For example, an IDR with 30 pathogenic *missense* mutations and 28 population *missense* mutations will *not* be categorized as *missense* mutation-intolerant as it complies with the first two criteria but not the third one and has an above-median population missense mutation. The list of all mutation-intolerant (n = 34) and tolerant (n = 533) disordered regions along with the frequency counts of different types of mutations in these regions are given in **S5 Table**.

### Statistical analysis

The two-sided Fisher’s Exact test of association was performed for each of the twenty-five UniProt features, taking the counts of disordered and non-annotated residues with and without a feature, to quantify the burden of each feature in disordered or non-annotated regions of IDPs (**Fig 1B**). An estimate of enrichment or burden (odds ratio, OR), 95% confidence interval (CI) of the OR value, and the p-value showing the significance of the observed burden or association, were obtained from the test output. All p-values (*p*) were corrected to generate “*q*” values, calculated as *p* × 25 according to the Bonferroni correction for multiple testing in statistical analysis. Therefore, a feature is considered to be a characteristic feature of disordered regions (DR feature) when the test outputs OR > 1 and *q* < 0.05. In contrast, when the test outputs OR < 1 and *q* < 0.05, the feature is referred to as a characteristic feature of non-annotated regions (NR feature). This approach of characterizing the disordered (and non-annotated) regions by comparative enrichment analysis taking both residue types into account in the two-tailed Fisher’s Exact test controls the possibility of obtaining a result simply because of the abundance of a certain feature in disordered or non-annotated regions of the protein. The same OR enrichment analysis was also performed on the “mutation-intolerant” and “mutation-tolerant” disordered regions to identify characteristics features of these two classes of IDRs (**Fig 5A**).

### Measuring relative features importance

The relative importance of UniProt features in predicting mutation-intolerant versus mutation-tolerant IDRs was measured using the “permutation feature importance” method. In this method, the increase in the prediction error or the decrease in the prediction accuracy of the classifier model is measured after the features’ values are randomly shuffled. The random shuffling of the values of a feature breaks the relationship between the feature and the true outcome, if any, and hence identifies the features that contribute the most to the predictive power of the classifier model. Additionally, the “permutation feature importance” method allows for determining the feature importance in a classification algorithm-agnostic fashion, as only the difference in the error or accuracy of the model is tracked. Here we used random forest algorithm to build the classifier model and evaluated the relative importance of twenty-five UniProt features in classifying “mutation-intolerant” versus “mutation-tolerant” disordered regions. Specifically, we fed the frequency counts of the features for each IDR into the classifier (i.e., number of “region of interest”, “modified residues”, etc. located in each IDR, **S6 Table**). The parameters of the classifier were set to: number of estimators or decision trees = 100 and quality measure = “gini” (**S7 Table**; summary of the analysis according to DOME: Data, Optimization, Model, Evaluation [73]). We repeated the permutation for 10 times and computed the mean decrease in the prediction score (i.e., average-precision, **Fig 5B**) generated by the model. Average precision score summarizes a precision-recall curve as the weighted mean of precisions achieved at each threshold, with the increase in recall from the previous threshold used as the weight and is well suited for assessing binary classification tasks. A feature is important if shuffling its values decreases the average precision of the model, otherwise it is unimportant. The classifier model and the feature importance evaluation method were implemented using the scikit-learn machine learning library for Python (https://scikit-learn.org/dev/).

## Supporting information

S1 Fig Distribution of frequency of residues (y-axis) annotated with twenty-five UniProt features (x-axis) in disordered regions (IDRs, count = 278) with experimentally verified disorder functions. Distributions are drawn separately for groups of IDRs that perform a specific function. To ensure the clarity of the visual, IDRs with less than 50 residues annotated with a feature were considered for the plot.
(TIF)

S2 Fig Distribution of frequency of residues (y-axis) annotated with twenty-five UniProt features (x-axis) in disordered regions (IDRs, count = 360) with experimentally verified interaction partners. Distributions are drawn separately for groups of IDRs that interact with a type of molecule. To ensure the clarity of the visual, IDRs with less than 50 residues annotated with a feature were considered for the plot.
(TIF)

S3 Fig Distribution of frequency of residues (y-axis) annotated with twenty-five UniProt features (x-axis) in disordered regions (IDRs, count = 190) with experimentally verified structural transitions. Distributions are drawn separately for groups of IDRs that undergo transitions from disorder to order and from order to disorder state. To ensure the clarity of the visual, IDRs with less than 50 residues annotated with a feature were considered for the plot.
(TIF)

S4 Fig Distribution of frequency of residues (y-axis) annotated with twenty-five UniProt features (x-axis) in disordered regions (IDRs, count = 981) with experimentally verified structural states. Distributions are drawn separately for groups of IDRs in disorder and order states. To ensure the clarity of the visual, IDRs with less than 50 residues annotated with a feature were considered for the plot.
(TIF)

S5 Fig Illustration of mutation-intolerant IDRs with their characteristic UniProt features for two intrinsically disordered proteins: MECP2 and DDX3X. (*A*) The methyl-CpG-binding protein 2 (MECP2) contains a nucleic acid-binding IDR (207–310), which functions as an inhibitor, and is identified as a mutation-intolerant IDR (**Table 1**). This IDR has UniProt features: “regions of interest” that interact with NCOR2, TBL1XR1, “modified residues” (phosphoserines), and “DNA binding region”; all these features are identified as the characteristic features of mutation-intolerant IDRs, in this study (**Fig 5**). This MECP2 IDR is associated with 77 frameshift, 13 nonsense, 14 missense and 4 inframe pathogenic mutations causing many neurodevelopmental disorders, according to the ClinVar database (**S5 Table**). (*B*) The ATP-dependent RNA helicase, DDX3X contains a 167-residues long mutation-intolerant IDR (**Table 1**). This IDR has no function annotation in the DisProt database (**Table 1**), but we observed seven “regions of interest”, interacting with multiple partners, and many “modified residues” (PTM sites) in this IDR, hinting to its function (protein-protein interaction, PTM-mediated signaling, etc.). Variations in this IDR (7 frameshift, 6 stop-gained, and 5 missense mutations) are associated with mental retardation and intellectual disability (**S5 Table**).
(TIF)

S6 Fig Characterization of the UniProt feature “motif” located in the disordered regions (IDRs). Out of 561 intrinsically disordered proteins (IDP) studied in this work, 143 proteins had at least one short linear motif (total count = 237) according to the UniProt database (referred to as UniProt feature: “motif”). 68 out of these 237 UniProt-annotated motifs are recorded in the Eukaryotic Linear Motif (ELM) resource, where they are grouped into different “ELM types” based on their functions. (*A*) Pi-chart showing the proportion of UniProt-annotated motifs located in IDRs of different ELM types. The most common type of motifs found in IDRs is LIG or ligand sites (41%), which mediate binding between the protein, harboring the ligand motif, and its interaction partner. (B) Proportion of motifs present in IDRs according to Gene Ontology (GO) terms, describing whether the motif is involved in biological processes (DNA repair/replication/damage, cell division/death, etc), molecular functions (e.g., growth factor receptor binding, phosphatase inhibitor activity, ubiquitin protein ligase binding), or is a cellular component (cytosol, nucleoplasm, etc.). Both charts correspond to 68 motifs that were observed as UniProt features in IDRs and were also annotated in the ELM resource with ELM types and GO terms (**S8 Table**).
(TIF)

S7 Fig Distribution of ratios of missense to synonymous (nmis/nsyn) variations from gnomAD database, representing genetic variants from relatively healthy individuals in the general population, in all IDRs (n = 945), and short (≤ 30 residues; n = 450), medium-length (30 < residues ≤ 100; n = 306) and long (>100 residues; n = 189) IDRs. (*A*) On average, the n_mis_/n_syn_ for all IDRs was 2.3 ± 1.4 (i.e. mean ± standard deviation), showing that regardless of length, IDRs carry over twice as many amino acid-substituting missense variations as synonymous variations. (*B*) Results for all IDRs consistently hold for long IDRs with a relatively low standard deviation (n_mis_/n_syn_ = 2.1 ± 0.6, minimum and maximum missense variation count per long IDR = 8 and 1265, respectively; in *green*). However, short IDRs display a wide variety (n_mis_/n_syn_ = 2.4 ± 1.8, minimum and maximum missense variation count per short IDR = 0 and 157, respectively; in *violet*). Specifically, we found 47 short IDRs that carry over five times more missense variations than synonymous variations (n_mis_/n_syn_ > = 5.0). At the same time, 38 short IDRs carried less than or equal to one-half number of missense variations as synonymous variations (n_mis_/n_syn_ < = 0.5). Out of these 38 short IDRs, 10 disordered regions in seven proteins (GTP-binding nuclear protein Ran, NF-kappa-B essential modulator, High mobility group protein B1, etc.) were entirely depleted of missense variations (count = 0), indicating that amino acid substitutions are likely not tolerated in these IDRs. Data corresponding to these plots are available in **S4 Table**.
(TIF)

S1 Table List of disordered regions (IDR, n = 981) in 561 intrinsically disordered proteins (IDPs) analyzed in this study. For each IDR, the table records the DisProt identifier and UniProtKB identifier for corresponding IDP, name of the gene encoding the IDP, start and end positions and length of the disordered region, and the annotation of function, interaction partner, structural state, and structural transition of the IDR, when available in the DisProt database.
(XLSX)

S2 Table Counts of disordered regions (IDRs) of different categories according to their disorder functions, interaction partners, structural transition, and structural states. The annotations are collected for 981 IDRs of 561 human intrinsically disordered proteins from the DisProt database.
(DOCX)

S3 Table Counts of genetic variations located in the disordered and non-annotated regions of intrinsically disorder proteins studied in this paper. Genetic variations found in the general population and patients are collected from gnomAD and ClinVar databases, respectively.
(DOCX)

S4 Table The frequency counts and ratio of amino acid substituting missense mutations and silent synonymous mutations in disordered regions. The table also reports the DisProt identifier for each disordered region (IDR), the gene encoding for the protein with the corresponding IDR, and the length of each IDR. An IDR with n_miss/n_syn < 1.0 harbors a lower frequency of missense mutations than synonymous mutations and an IDR with n_mis/n_syn > 1.0 harbors a higher frequency of missense mutations than synonymous mutations. Distributions of n_mis/n_syn for IDRs of different lengths are shown in **Fig 3** (main text) and **S7 Fig** (supplemental).
(XLSX)

S5 Table List of mutation-intolerant (n = 34) and mutation-tolerant (n = 533) disordered regions (IDRs) identified in this study. For each IDR, the table lists the DisProt identifier, name of the gene encoding for the IDP, length of the disordered region, and the annotation of disorder function, interaction partner, structural state, and structural transition of the IDR, when available in the DisProt database. Further, we report the number of “pathogenic” (ClinVar database) and “population” (gnomAD database) mutations that are located in each IDR, followed by the diseases associated with the germline pathogenic mutations affecting the IDR.
(XLSX)

S6 Table Frequency counts of UniProt features in “mutation-intolerant” and “mutation-tolerant” IDRs (see the last column for the annotation of IDRs). This feature set were fed into the random forest classifier to compute the permutation importance of each feature in classifying “mutation-intolerant” versus “mutation-tolerant” IDRs (see results in Fig 5B).
(XLSX)

S7 Table Summary table for ML analysis performed to measure relative importance of UniProt features in stratifying “mutation-intolerant” versus “mutation-tolerant” disordered regions (IDRs), according to DOME (Data, Optimization, Model, Evaluation): Recommendations for supervised machine learning validation in biology. (DOCX)

S8 Table List of UniProt feature "motif" (n = 68) in disordered regions (IDRs) that were found in the Eukaryotic Linear Motif (ELM) resource. Out of 561 intrinsically disordered proteins (IDPs) studied in this work, 143 proteins had at least one short linear motif (total count = 237) according to the UniProt database (referred to as UniProt feature: “motif”). 68 out of these 237 UniProt-annotated motifs are recorded in the ELM resource, where they are grouped into different “ELM types” based on their function. For each of these 68 motifs, the table lists the UniProt feature description, UniProt identifier, gene name, ELM accession, identifier, type, and the start/end position of the motif as recorded in ELM. Additionally, we report the Gene Ontology terms for each motif as available in the ELM resource. The possible ELM types are: LIG—ligand sites, DOC—docking sites, TRG—subcellular targeting sites, DEG—degradation sites, and MOD—PTM sites. The proportion of motifs in different ELM types and GO terms are shown in **S6 Fig**.
(XLSX)

S9 Table List of UniProt feature "domain" (n = 124) that overlap with disordered regions (IDRs) of 105 intrinsically disordered proteins. For each domain, the table lists the UniProt feature description (UniProt feature: domain), UniProt identifier, gene name, DisProt identifier for the protein, start/end of the disordered regions that overlap with the domain, categories of IDRs in terms of their function, interaction partners, structural state, and structural transitions (DisProt annotation). Additionally, we report whether a UniProt-annotated domain is present in the Disordered Binding Sites (DIBS) and the Mutual Folding Induced by Binding (MFIB) databases. When a domain is present in these databases, we report the accession of the corresponding entry in the database and the PDB ID reported in these databases, as a form of evidence. 30 out of 124 UniProt-annotated domains have been determined to have the “disorder” structural state and undergo “disorder to order” structural transition, according to DisProt (highlighted in orange). Eleven UniProt-annotated domains, overlapping with IDRs, were also annotated in DIBS and MIFB databases, highlighted in green and yellow, respectively, to being unstructured in isolation but forming structures only upon binding.
(XLSX)
