# Supplementary figures and images for: Characterization of intrinsically disordered regions in proteins informed by human genetic diversity
Source: PLoS Comput Biol. 2022 Mar 11;18(3):e1009911. doi: 10.1371/journal.pcbi.1009911 (PMC8942211; doi:10.1371/journal.pcbi.1009911)

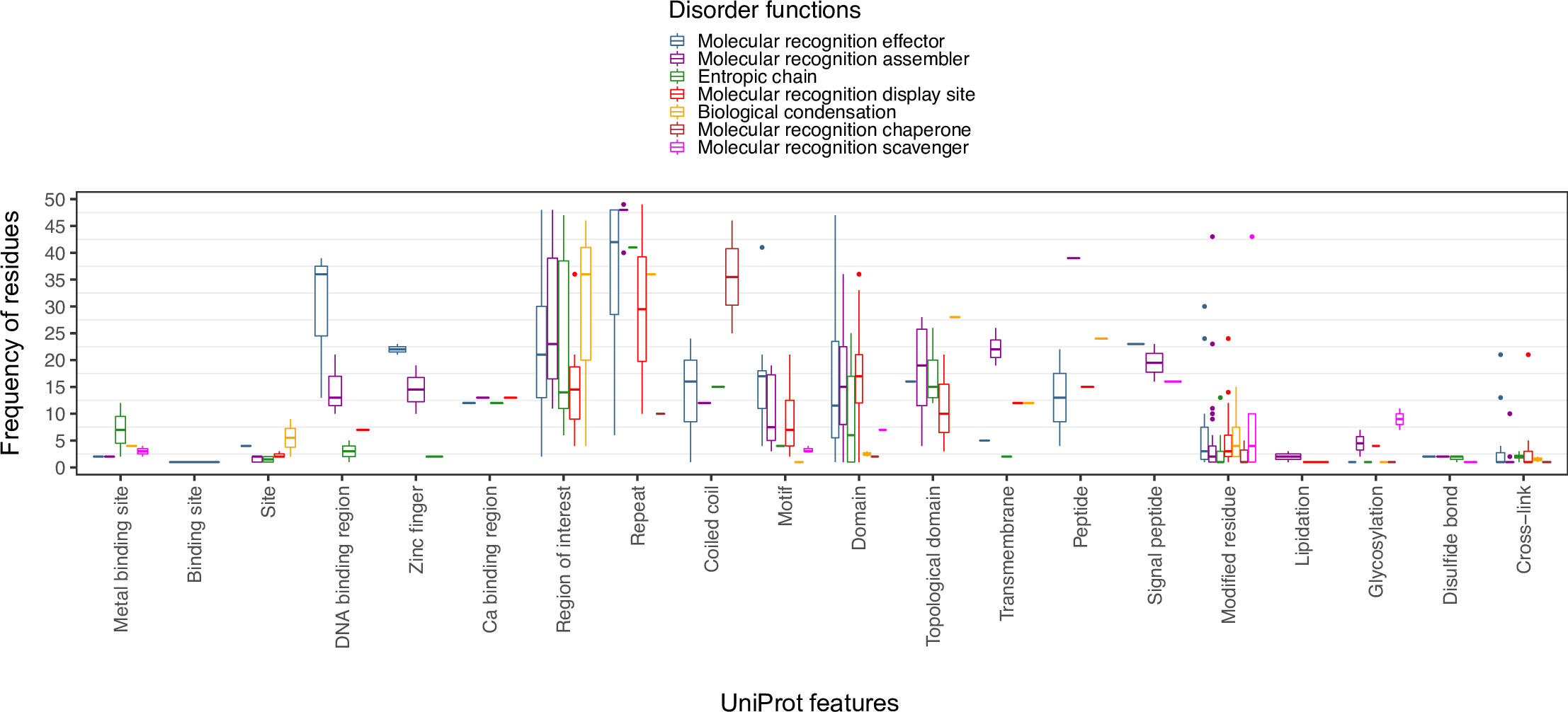

Supplement: S1 Fig — Distributions are drawn separately for groups of IDRs that perform a specific function. To ensure the clarity of the visual, IDRs with less than 50 residues annotated with a feature were considered for the plot. (TIF) [file pcbi.1009911.s001.tif]

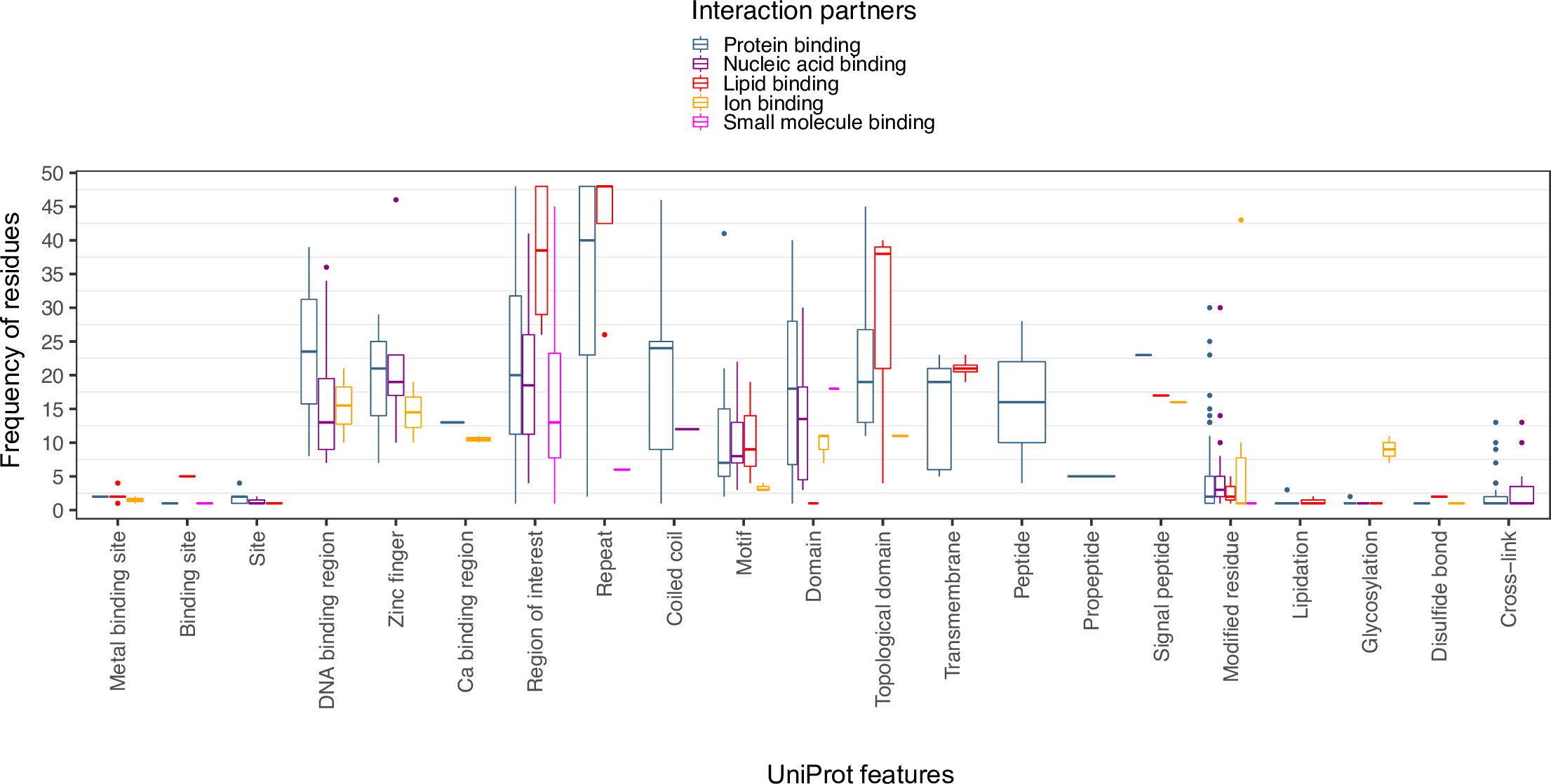

Supplement: S2 Fig — Distributions are drawn separately for groups of IDRs that interact with a type of molecule. To ensure the clarity of the visual, IDRs with less than 50 residues annotated with a feature were considered for the plot. (TIF) [file pcbi.1009911.s002.tif]

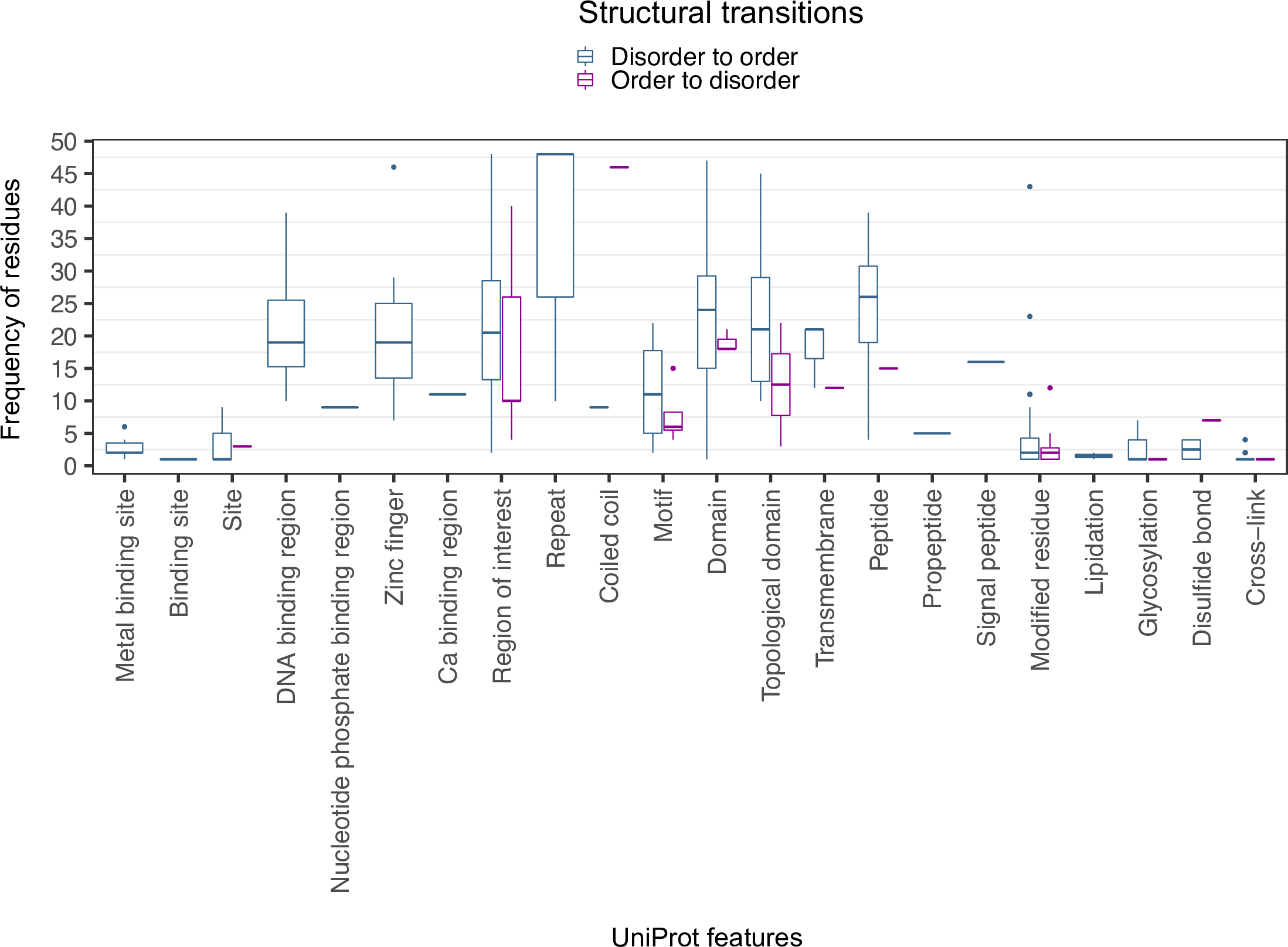

Supplement: S3 Fig — Distributions are drawn separately for groups of IDRs that undergo transitions from disorder to order and from order to disorder state. To ensure the clarity of the visual, IDRs with less than 50 residues annotated with a feature were considered for the plot. (TIF) [file pcbi.1009911.s003.tif]

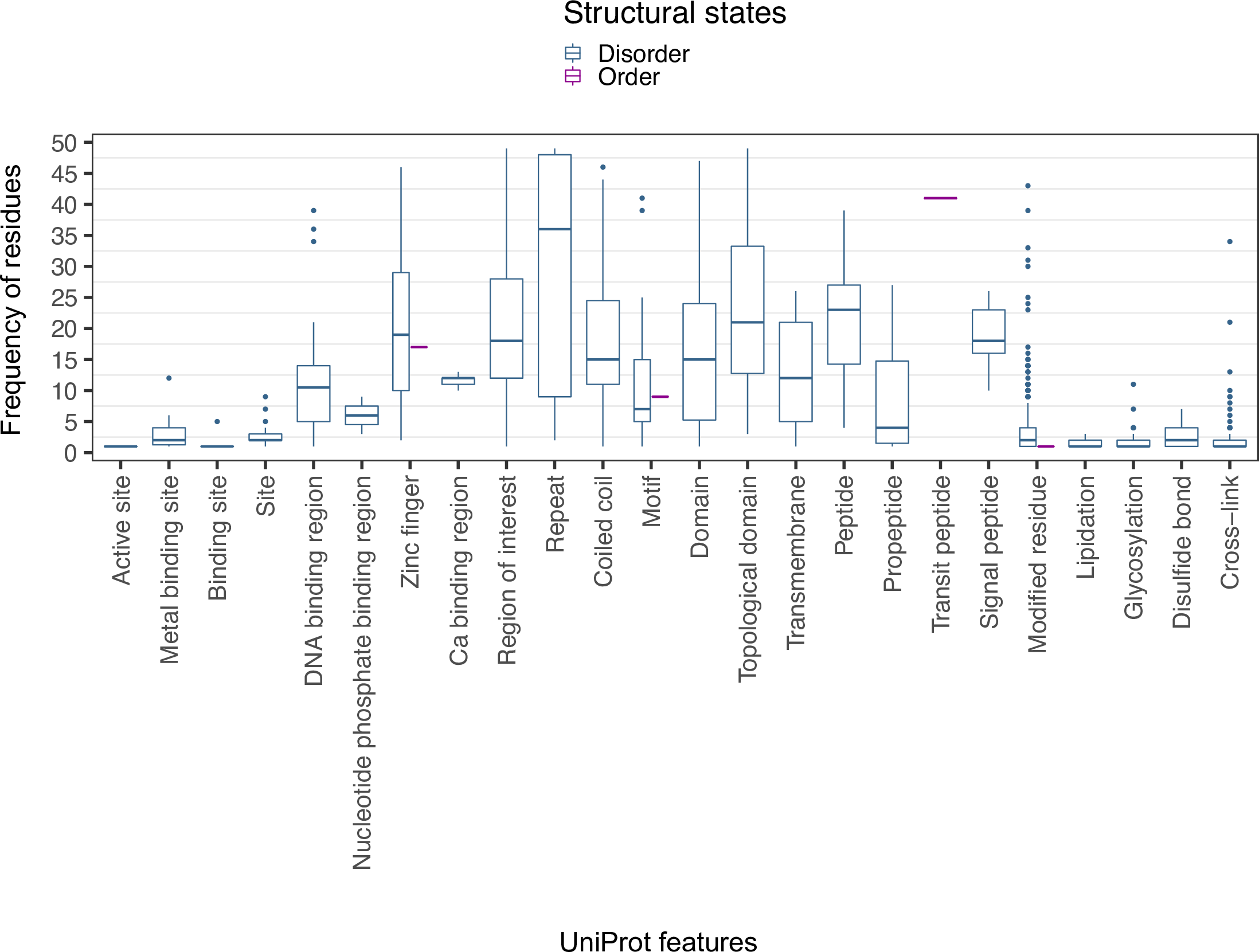

Supplement: S4 Fig — Distributions are drawn separately for groups of IDRs in disorder and order states. To ensure the clarity of the visual, IDRs with less than 50 residues annotated with a feature were considered for the plot. (TIF) [file pcbi.1009911.s004.tif]

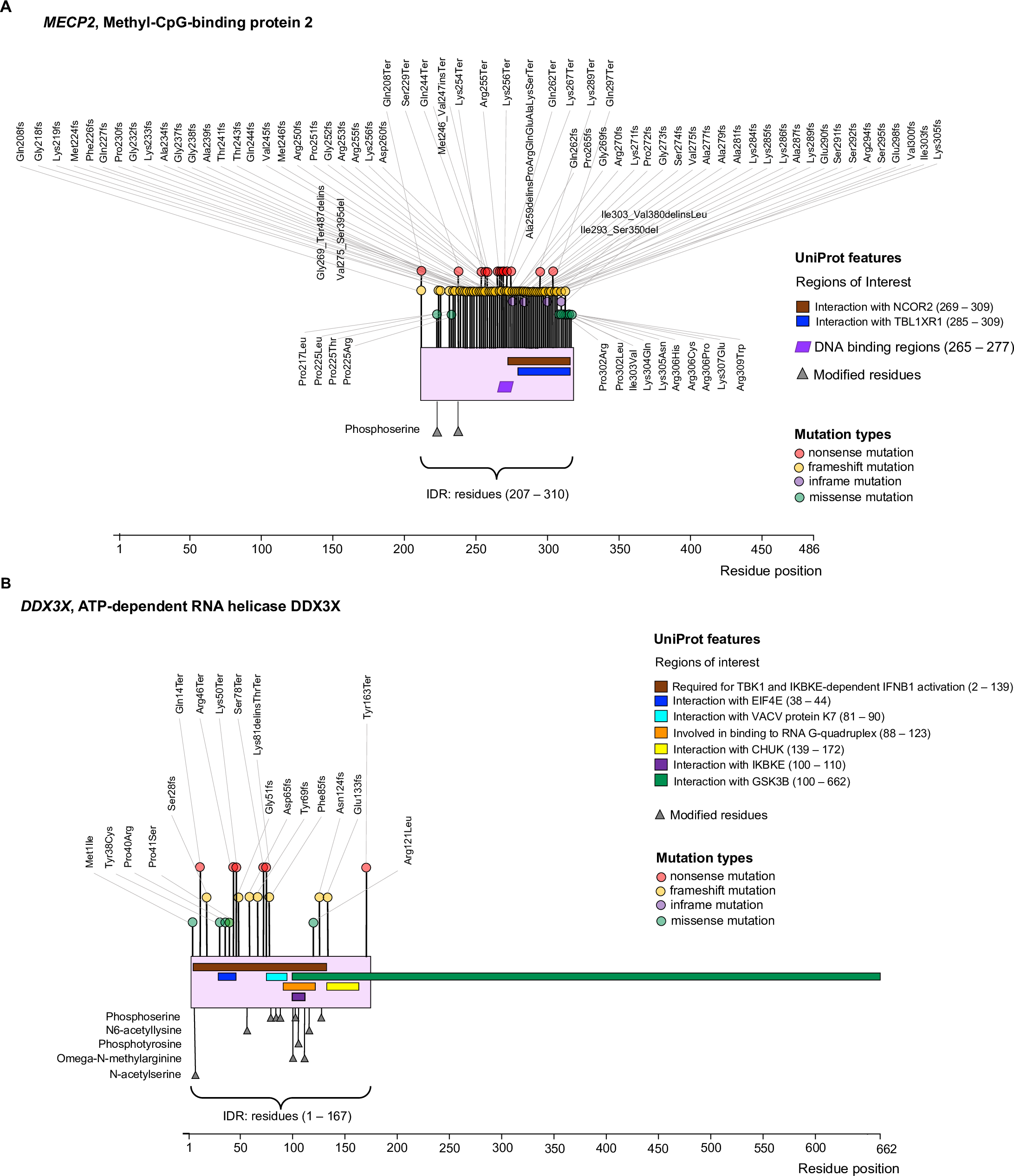

Supplement: S5 Fig — (A) The methyl-CpG-binding protein 2 (MECP2) contains a nucleic acid-binding IDR (207–310), which functions as an inhibitor, and is identified as a mutation-intolerant IDR (Table 1). This IDR has UniProt features: “regions of interest” that interact with NCOR2, TBL1XR1, “modified residues” (phosphoserines), and “DNA binding region”; all these features are identified as the characteristic features of mutation-intolerant IDRs, in this study (Fig 5). This MECP2 IDR is associated with 77 frameshift, 13 nonsense, 14 missense and 4 inframe pathogenic mutations causing many neurodevelopmental disorders, according to the ClinVar database (S5 Table). (B) The ATP-dependent RNA helicase, DDX3X contains a 167-residues long mutation-intolerant IDR (Table 1). This IDR has no function annotation in the DisProt database (Table 1), but we observed seven “regions of interest”, interacting with multiple partners, and many “modified residues” (PTM sites) in this IDR, hinting to its function (protein-protein interaction, PTM-mediated signaling, etc.). Variations in this IDR (7 frameshift, 6 stop-gained, and 5 missense mutations) are associated with mental retardation and intellectual disability (S5 Table). (TIF) [file pcbi.1009911.s005.tif]

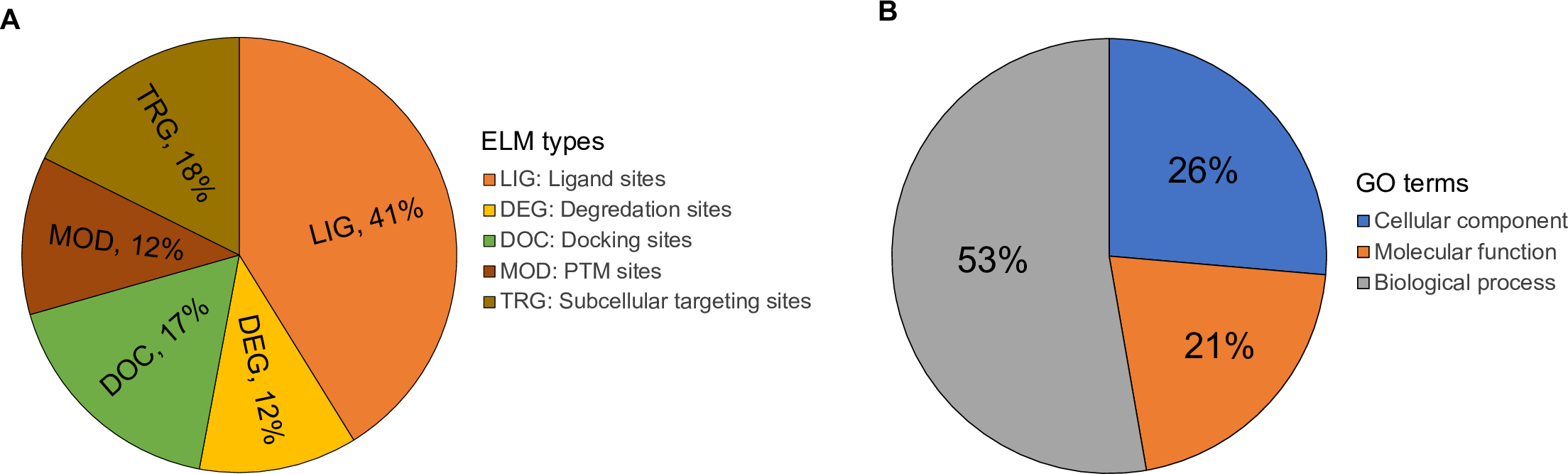

Supplement: S6 Fig — Out of 561 intrinsically disordered proteins (IDP) studied in this work, 143 proteins had at least one short linear motif (total count = 237) according to the UniProt database (referred to as UniProt feature: “motif”). 68 out of these 237 UniProt-annotated motifs are recorded in the Eukaryotic Linear Motif (ELM) resource, where they are grouped into different “ELM types” based on their functions. (A) Pi-chart showing the proportion of UniProt-annotated motifs located in IDRs of different ELM types. The most common type of motifs found in IDRs is LIG or ligand sites (41%), which mediate binding between the protein, harboring the ligand motif, and its interaction partner. (B) Proportion of motifs present in IDRs according to Gene Ontology (GO) terms, describing whether the motif is involved in biological processes (DNA repair/replication/damage, cell division/death, etc), molecular functions (e.g., growth factor receptor binding, phosphatase inhibitor activity, ubiquitin protein ligase binding), or is a cellular component (cytosol, nucleoplasm, etc.). Both charts correspond to 68 motifs that were observed as UniProt features in IDRs and were also annotated in the ELM resource with ELM types and GO terms (S8 Table). (TIF) [file pcbi.1009911.s006.tif]

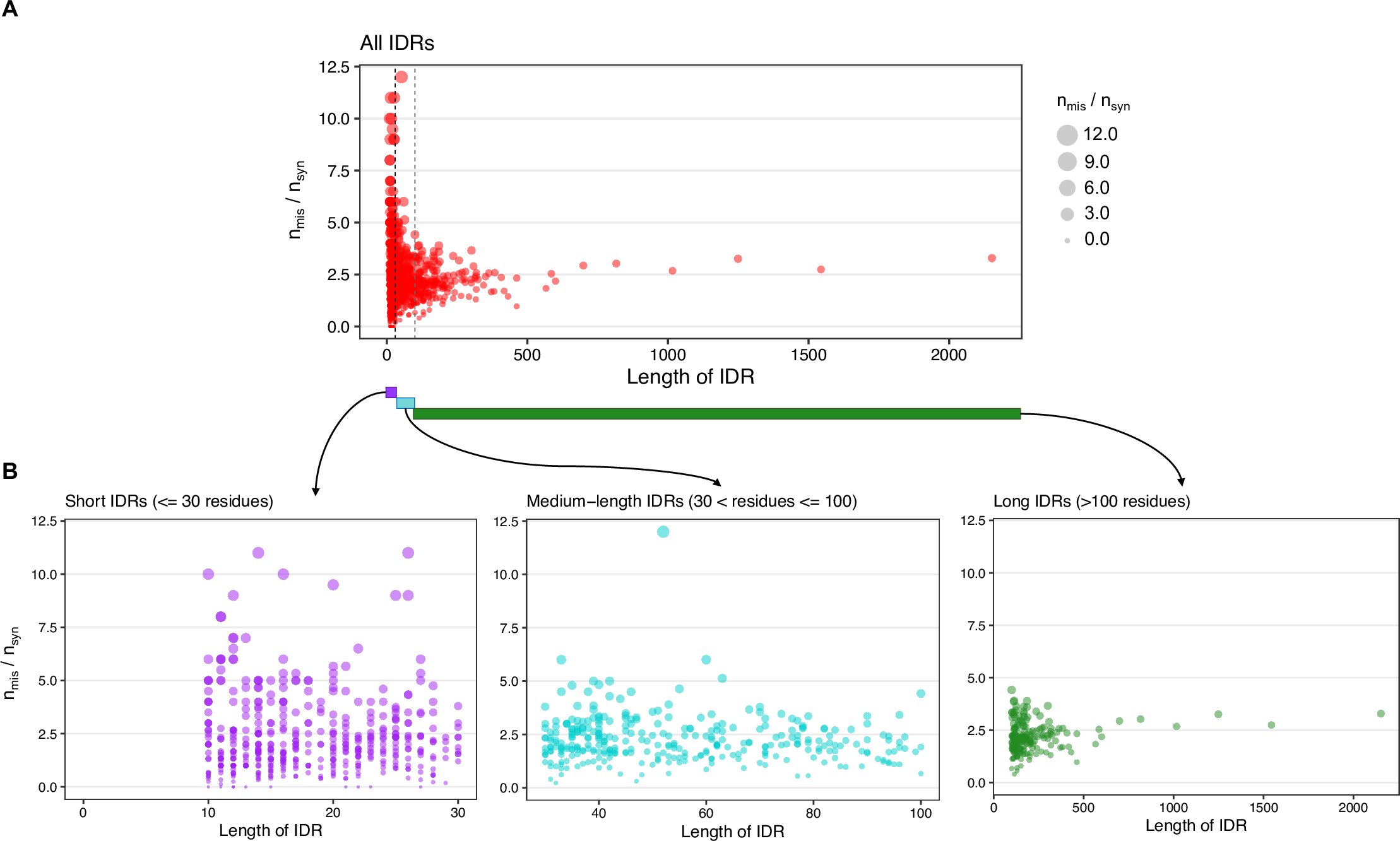

Supplement: S7 Fig — (A) On average, the nmis/nsyn for all IDRs was 2.3 ± 1.4 (i.e. mean ± standard deviation), showing that regardless of length, IDRs carry over twice as many amino acid-substituting missense variations as synonymous variations. (B) Results for all IDRs consistently hold for long IDRs with a relatively low standard deviation (nmis/nsyn = 2.1 ± 0.6, minimum and maximum missense variation count per long IDR = 8 and 1265, respectively; in green). However, short IDRs display a wide variety (nmis/nsyn = 2.4 ± 1.8, minimum and maximum missense variation count per short IDR = 0 and 157, respectively; in violet). Specifically, we found 47 short IDRs that carry over five times more missense variations than synonymous variations (nmis/nsyn > = 5.0). At the same time, 38 short IDRs carried less than or equal to one-half number of missense variations as synonymous variations (nmis/nsyn < = 0.5). Out of these 38 short IDRs, 10 disordered regions in seven proteins (GTP-binding nuclear protein Ran, NF-kappa-B essential modulator, High mobility group protein B1, etc.) were entirely depleted of missense variations (count = 0), indicating that amino acid substitutions are likely not tolerated in these IDRs. Data corresponding to these plots are available in S4 Table. (TIF) [file pcbi.1009911.s007.tif]
