# Supplementary material for: Characterization of intrinsically disordered regions in proteins informed by human genetic diversity
Source: PLoS Comput Biol. 2022 Mar 11;18(3):e1009911. doi: 10.1371/journal.pcbi.1009911 (PMC8942211; doi:10.1371/journal.pcbi.1009911)
Supplement: S2 Table — The annotations are collected for 981 IDRs of 561 human intrinsically disordered proteins from the DisProt database. (DOCX) [file pcbi.1009911.s009.docx]

**S2 Table**. Counts of disordered regions (IDRs) of different categories according to their disorder functions, interaction partners, transition states and structural states. The annotations are collected for 981 IDRs of 561 human intrinsically disordered proteins from the DisProt database.

| Categories | Category names | IDR counts |
| --- | --- | --- |
| Disorder functions | Total known | 278 |
|  | Molecular recognition effector | 86 |
|  | Molecular recognition assembler | 80 |
|  | Entropic chain | 69 |
|  | Molecular recognition display site | 48 |
|  | Biological condensation | 12 |
|  | Molecular recognition chaperone | 9 |
|  | Molecular recognition scavenger | 7 |
|  | Unknown disorder function | 703 |
|  |  |  |
| Interaction partners | Total known | 360 |
|  | Protein binding | 287 |
|  | Nucleic acid binding | 58 |
|  | Lipid binding | 14 |
|  | Ion binding | 13 |
|  | Small molecule binding | 10 |
|  | Unknown interaction partner | 621 |
|  |  |  |
| Transition states | Total known | 190 |
|  | Disorder to order | 165 |
|  | Order to disorder | 25 |
|  | Unknown transition state | 791 |
|  |  |  |
| Structural state | Total known | 981 |
|  | Disorder | 978 |
|  | Order | 3 |
|  | Unknown structural state | 0 |
|  |  |  |
| Total IDRs |  | 981 |
