# Supplementary material for: Characterization of intrinsically disordered regions in proteins informed by human genetic diversity
Source: PLoS Comput Biol. 2022 Mar 11;18(3):e1009911. doi: 10.1371/journal.pcbi.1009911 (PMC8942211; doi:10.1371/journal.pcbi.1009911)
Supplement: S3 Table — Genetic variations found in the general population and patients are collected from gnomAD and ClinVar databases, respectively. (DOCX) [file pcbi.1009911.s010.docx]

S3 Table. Counts of genetic variations located in the disordered and non-annotated regions of intrinsically disorder proteins studied in this paper. Genetic variations found in the general population and patients are collected from gnomAD [1] and ClinVar [2] databases, respectively.

| **gnomAD database (548 proteins)** | | | | | | | |
| --- | --- | --- | --- | --- | --- | --- | --- |
|  | Disordered regions | | | Not-annotated regions | | | Total |
| Variation or mutation type | All | Allele count  = 1 | Allele count  > 1 | All | Allele count = 1 | Allele count > 1 |  |
| Missense | 41691 | 21436 | 17660 | 176888 | 92106 | 75266 | 218579 |
| Nonsense* | 955 | 555 | 276 | 4117 | 2403 | 1139 | 5072 |
| Frameshift* | 1814 | 1052 | 526 | 6113 | 4029 | 1368 | 7927 |
| Inframe* (insertions/deletions) | 1828 | 841 | 863 | 4042 | 1970 | 1794 | 5870 |
| Synonymous | 20282 | 9076 | 10017 | 92314 | 42223 | 45702 | 112596 |
| **ClinVar database (96 proteins)** | | | | | | | |
|  | Disordered regions | | | Not-annotated regions | | | Total |
| Variation or mutation type | Pathogenic^+^ | Benign^+^ | Uncertain significance | Pathogenic^+^ | Benign^+^ | Uncertain significance |  |
| Missense | 230 | 189 | 2678 | 1742 | 475 | 7512 | 12826 |
| Nonsense* | 836 | 0 | 25 | 1902 | 6 | 32 | 2801 |
| Frameshift* | 1606 | 0 | 22 | 3553 | 12 | 41 | 5234 |
| Inframe* (insertions/deletions) | 59 | 27 | 158 | 208 | 25 | 330 | 807 |

*Inframe deletions or insertions usually affect multiple amino acids, and frameshift and nonsense mutations often result in completely altered, shorter or unfinished protein products. For these variation types, counts reported in the table refer to the number of mutations for which the first amino acid affected by the mutation is located in a disordered or non-annotated region.

+ Pathogenic and likely-pathogenic variations are jointly referred to as “pathogenic”. Similarly, benign and likely-benign variations are jointly referred to as “benign”.

**References**

1. Karczewski KJ, Francioli LC, Tiao G, Cummings BB, Alfoldi J, Wang Q, et al. The mutational constraint spectrum quantified from variation in 141,456 humans. Nature. 2020;581(7809):434-43. Epub 2020/05/29. doi: 10.1038/s41586-020-2308-7. PubMed PMID: 32461654.

2. Landrum MJ, Lee JM, Benson M, Brown GR, Chao C, Chitipiralla S, et al. ClinVar: improving access to variant interpretations and supporting evidence. Nucleic Acids Res. 2018;46(D1):D1062-D7. Epub 2017/11/23. doi: 10.1093/nar/gkx1153. PubMed PMID: 29165669; PubMed Central PMCID: PMCPMC5753237.
