# Supplementary material for: Characterization of intrinsically disordered regions in proteins informed by human genetic diversity
Source: PLoS Comput Biol. 2022 Mar 11;18(3):e1009911. doi: 10.1371/journal.pcbi.1009911 (PMC8942211; doi:10.1371/journal.pcbi.1009911)
Supplement: S7 Table — (DOCX) [file pcbi.1009911.s014.docx]

**S7 Table.** Summary table for ML analysis performed to measure relative importance of UniProt features in stratifying “mutation-intolerant” versus “mutation-tolerant” disordered regions (IDRs), according to DOME (Data, Optimization, Model, Evaluation): Recommendations for supervised machine learning validation in biology [1].

| **DOME** | Version | 1.0 |
| --- | --- | --- |
| **Data** | Provenance | Database of Protein Disorder (DisProt), version 8.0.2, release 2020_06. N_pos_ = 34 (mutation-intolerant IDRs), N_neg_ = 533 (mutation-tolerant IDRs). The identification of mutation-intolerant and mutation-tolerant IDRs has been performed as part of this study. |
|  | Dataset splits | No |
|  | Redundancy between data splits | No |
|  | Availability of data | Yes: supplementary table (S6 Table) |
| **Optimization** | Algorithm | Random forest |
|  | Meta-predictions | No |
|  | Data encoding | Global features, no sliding windowing. |
|  | Parameters | Number of estimators or decision trees  Criterion or function to measure the quality of split |
|  | Features | **25 UniProt features (f = 25):** the frequency counts of features for each IDR (i.e., number of “region of interest”, “modified residues”, etc. located in each IDR.  **Protocol**: permutation feature importance (<https://scikit-learn.org/stable/modules/permutation_importance.html>). Permutation (random shuffling to break the relationship between the feature and the outcome) of each feature’s values was repeated for 10 times to compute the “mean decrease in average-precision”—as the importance of that feature in stratifying mutation-intolerant versus mutation-tolerant IDRs—of the classifier model, after permuting the feature’s values. This method allows for determining the feature importance in a classification algorithm agnostic fashion, as only the difference in the error or accuracy of the model is tracked.  Feature importance was estimated on training data only. |
|  | Fitting | No |
|  | Regularization | No |
|  | Availability of configuration | Number of estimators or decision trees = 100  Criterion or function to measure the quality of split = “gini” |
| **Model** | Interpretability | Black box |
|  | Output | Classification, to ultimately measure feature importance. |
|  | Execution time | 3 – 5 mins |
|  | Availability of software | GitHub: https://github.com/iqbals/Perm_feat_importance |
| **Evaluation** | Evaluation method | Not applicable |
|  | Performance measure | Average precision |
|  | Confidence | Random shuffling of feature values was repeated for 10 times to compute standard deviation |
|  | Availability of evaluation | No. |

References

1. Walsh I, Fishman D, Garcia-Gasulla D, Titma T, Pollastri G, Group EMLF, et al. DOME: recommendations for supervised machine learning validation in biology. Nat Methods. 2021;18(10):1122-7. Epub 2021/07/29. doi: 10.1038/s41592-021-01205-4. PubMed PMID: 34316068.
